# Combination of AFP vaccine and immune checkpoint inhibitors slows hepatocellular carcinoma progression in preclinical models

**DOI:** 10.1172/JCI163291

**Published:** 2023-06-01

**Authors:** Xinjun Lu, Shanshan Deng, Jiejie Xu, Benjamin L. Green, Honghua Zhang, Guofei Cui, Yi Zhou, Yi Zhang, Hongwei Xu, Fapeng Zhang, Rui Mao, Sheng Zhong, Thorsten Cramer, Matthias Evert, Diego F. Calvisi, Yukai He, Chao Liu, Xin Chen

**Affiliations:** 1 Department of Biliary-Pancreatic Surgery, Sun Yat-sen Memorial Hospital, Sun Yat-sen University, Guangzhou, China.; 2 Department of Bioengineering and Therapeutic Sciences, University of California, San Francisco, California, USA.; 3 Department of Hearing and Speech Science, Guangzhou Xinhua University, Guangzhou, China.; 4 University of Hawaii Cancer Center, Honolulu, Hawaii, USA.; 5 Department of Infectious Diseases, The First Affiliated Hospital of Xi’an Jiaotong University, Xi’an, China.; 6 Key Laboratory of Biorheological Science and Technology, Ministry of Education, College of Bioengineering, Chongqing University, Chongqing, China.; 7 Department of Liver Surgery, Center of Liver Transplantation, West China Hospital of Sichuan University, Chengdu, China.; 8 Department of Medicine, Medical College of Georgia, Augusta University, Augusta, Georgia, USA.; 9 Department of General, Visceral and Transplantation Surgery, RWTH University Hospital, Aachen, Germany.; 10 Department of Surgery, Maastricht University Medical Center, Maastricht, The Netherlands.; 11 Institute of Pathology, University of Regensburg, Regensburg, Germany.

**Keywords:** Hepatology, Immunology, Immunotherapy, Liver cancer, T cells

## Abstract

Many patients with hepatocellular carcinoma (HCC) do not respond to the first-line immune checkpoint inhibitor treatment. Immunization with effective cancer vaccines is an attractive alternative approach to immunotherapy. However, its efficacy remains insufficiently evaluated in preclinical studies. Here, we investigated HCC-associated self/tumor antigen, α-fetoprotein–based (AFP-based) vaccine immunization for treating AFP (+) HCC mouse models. We found that AFP immunization effectively induced AFP-specific CD8^+^ T cells in vivo. However, these CD8^+^ T cells expressed exhaustion markers, including PD1, LAG3, and Tim3. Furthermore, the AFP vaccine effectively prevented c-MYC/Mcl1 HCC initiation when administered before tumor formation, while it was ineffective against full-blown c-MYC/Mcl1 tumors. Similarly, anti-PD1 and anti–PD-L1 monotherapy showed no efficacy in this murine HCC model. In striking contrast, AFP immunization combined with anti–PD-L1 treatment triggered significant inhibition of HCC progression in most liver tumor nodules, while in combination with anti-PD1, it induced slower tumor progression. Mechanistically, we demonstrated that HCC-intrinsic PD-L1 expression was the primary target of anti–PD-L1 in this combination therapy. Notably, the combination therapy had a similar therapeutic effect in the cMet/β-catenin mouse HCC model. These findings suggest that combining the AFP vaccine and immune checkpoint inhibitors may be effective for AFP (+) HCC treatment.

## Introduction

Hepatocellular carcinoma (HCC) accounts for most primary liver tumors and is one of the deadliest malignancies worldwide (1). Most HCCs are diagnosed in the advanced stage when limited therapeutic options are available. For years, the multikinase inhibitors sorafenib and lenvatinib represented the first-line drug regimens, although they only provided limited survival benefits to patients with progressed HCC (2). The success of immune checkpoint inhibitors (ICI), targeting programmed cell death protein 1 (PD1) and its ligand PD-L1 (also called CD274), and cytotoxic T-lymphocyte-associated protein 4 (CTLA4) in the therapy of some solid tumors has sparked significant interest in applying immunotherapy to HCC. Recently, the breakthrough of the Atezolizumab (anti–PD-L1) plus Bevacizumab (anti-VEGF) and tremelimumab (anti-CTLA4) plus durvalumab (anti-PD1) combination treatments in HCC demonstrated the promise of immunotherapy in the treatment of HCC (3, 4).

α-fetoprotein (AFP) is a major plasma protein produced by the yolk sac and the fetal liver during embryonic development. AFP level decreases rapidly after birth and is reexpressed in most HCC tumors, serving as a diagnostic biomarker in the clinic (5). The function of AFP in adults is unclear; nonetheless, it is a promising potential target for HCC immunotherapy (6–9). Butterfield et al. first identified 4 HLA-A*0201–restricted AFP epitopes and developed AFP peptide–based vaccinations for HCC (10, 11). This pioneering work suggested that AFP-recognizing T cells had not been entirely deleted from the T cell repertoire during the negative selection in the thymus. Since modest AFP-specific CD8^+^ T cell response was detected, these findings established a potential target for AFP-based HCC immunotherapy. A phase I/II clinical trial was conducted to evaluate the immunizing efficiency of these 4 AFP peptides pulsed onto dendritic cells (DC) in patients with HCC. Moderate AFP-specific T cell responses were detected to at least one of the peptides after vaccination (6). However, no significant clinical benefits were observed in these patients, suggesting that additional approaches may be needed to enhance this antitumor response.

Previously, a study reported multiple AFP epitopes that could bind to the mouse major histocompatibility complex–class I (MHC I) molecules based on an Epitoptimizer algorithm (12). Further amino acid modifications were employed to increase the affinity of the peptides to MHC I. The optimized AFP499 and AFP212 peptides induced high AFP antigen-specific T cells. Not surprisingly, followed by an AFP prime and boost immunization strategy, more infiltrating CD8^+^ T cells appeared in the tumors, and AFP499 and AFP212 peptides significantly delayed the subcutaneous and DEN-induced mouse HCC development (13). These studies suggested that the AFP vaccine could help prevent or delay HCC formation. However, whether this approach has therapeutic efficacy against existing HCC lesions remains unknown.

In the current study, we investigated the therapeutic potency of the AFP vaccination strategy (AFP lentivirus prime plus AFP499 peptide boost) in the c-MYC/Mcl1 and cMet/β-catenin murine HCC models. Our studies suggest that AFP immunization has limited antitumor activities against HCC. However, combined AFP vaccination and anti–PD-L1 antibody strongly inhibited HCC progression, supporting the usefulness of this combination immunotherapy for treating HCC.

## Results

### AFP immunization elicits functional AFP499^+^ cytotoxic T lymphocytes.

**Figure 1:**
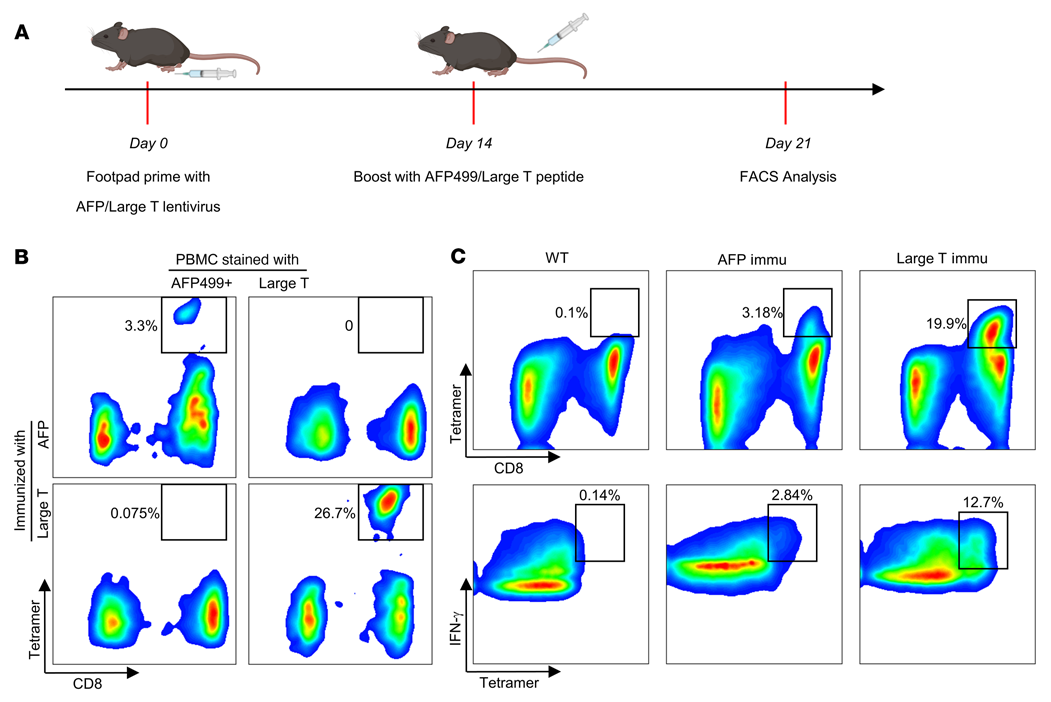
AFP or Large T antigen immunization elicits functional peptide-specific CD8^+^ T cells. (**A**) Overview of AFP and Large T peptide immunization procedure. (**B**) Representative results of AFP and Large T peptide-specific CD8^+^ T cells induced by the corresponding peptides. (**C**) Both AFP and Large T peptide-specific CD8^+^ T cells exhibit robust IFN-γ expression. Immu, immunization.

A previous study identified an H2-K^b^ restricted and optimized AFP peptide (AFP499) that could induce AFP499 peptide-specific CD8*^+^* cytotoxic T lymphocytes (CTLs) (AFP499*^+^* CTLs) by AFP lentivirus prime and AFP499 peptide boost strategy (Figure 1, A and B) (13). As a positive control, we used a similar approach to express a foreign antigen, namely the SV40 Large T antigen, which induced high levels of Large T peptide-specific CD8*^+^* T cells (Figure 1B). Importantly, no cross-reaction of AFP499 and Large T tetramer staining was detected, confirming their specificity. Notably, most peptide-specific CD8*^+^* T cells, induced by the AFP499 or Large T, expressed abundant IFN-γ (Figure 1C), a marker for immune cell activation, indicating these CD8*^+^* T cells were potentially functional. Other effects of the AFP vaccination strategy included increased CD8*^+^* T cells and decreased CD4*^+^* T cells and B cells in the liver (Supplemental Figure 1; supplemental material available online with this article; https://doi.org/10.1172/JCI163291DS1). In summary, the AFP lentivirus prime and AFP499 peptide boost strategy effectively induced AFP499*^+^* CTL in vivo.

### AFP immunization significantly delays autologous HCC initiation.

To evaluate whether AFP immunization can be effective against AFP(+) HCC, we applied the c-MYC/Mcl1–induced mouse HCC model by hydrodynamic tail vein injection (HDTVi), which exhibited high *Afp* expression in tumor cells (Supplemental Figure 2, A and B) (14–16). As the first step, we used Large T antigen as the control and asked if Large T immunization could prevent large T antigen (+) HCC development in vivo. Large T overexpression did not affect c-MYC/Mcl1–driven HCC formation (Supplemental Figure 3, A, B, and F). We immunized the mice with large T antigen and injected c-MYC/Mcl1 plasmids or c-MYC/Mcl1 together with pT3EF1α-Large T plasmid (c-MYC/Mcl1/Large T) (Supplemental Figure 3C). Noticeably, Large T immunization effectively prevented HCC development in c-MYC/Mcl1/Large T mice but not in c-MYC/Mcl1 mice (Supplemental Figures 3, C–F). The results suggested that immunization of tumor antigens might effectively prevent tumor initiation.

**Figure 2:**
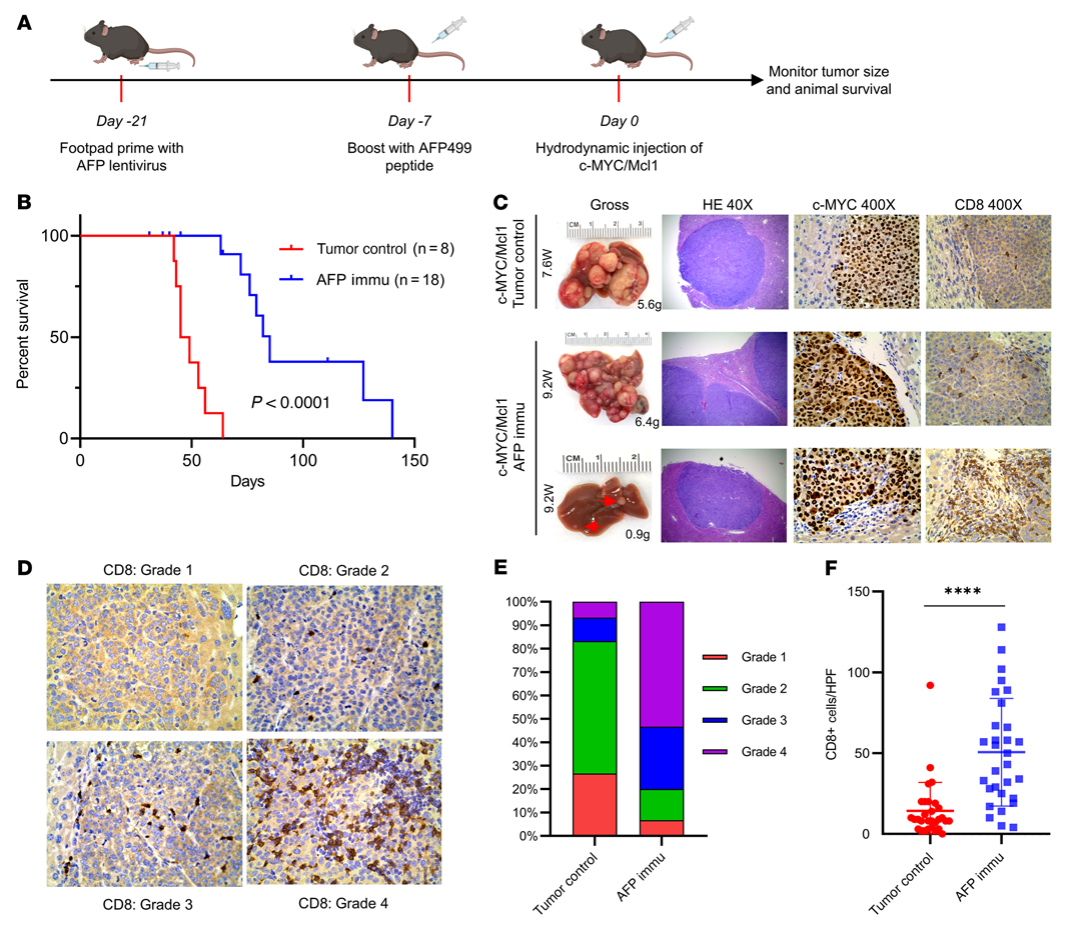
AFP immunization prevents the autologous HCC formation induced by c-MYC/Mcl1 hydrodynamic injection. (**A**) Study design of AFP immunization and c-MYC/Mcl1 hydrodynamic injection. (**B**) Survival curve of the tumor control and AFP immunization group in c-MYC/Mcl1 mouse model. (**C**) Representative gross images and histopathological features of HE, c-MYC, and CD8 staining in the tumor control and AFP immunized group. The numbers indicate the liver weight and weeks from plasmid injections to the sacrifice date for that particular mouse. Original magnification, 40 × and 400 ×, as indicated in the column head. (**D**) The intensity of CD8 staining in **C** was graded from 1–4 based on the defined infiltrated CD8^+^ T cell number; Grade 1: 0–5 cells; Grade 2: 6–20 cells; Grade 3: 21–40 cells; Grade 4: > 40 cells. Original magnification, 400 ×. (**E**) The infiltrated CD8^+^ T cells were quantified in the tumor control and AFP-immunized groups (*n* = 30 high power fields). Kaplan-Meier test was used for survival analysis in **B**, unpaired 2-tailed Student’s *t* test was used in (**F**). Data are presented as mean ± SD. ****P < 0.0001. W, week(s); HPF, high power field; Immu, immunization.

Next, we asked whether AFP immunization could prevent c-MYC/Mcl1-driven HCC initiation. We primed the mice with AFP lentivirus and then boosted them with AFP499 peptide 2 weeks later. After the AFP peptide-specific CD8^+^ T cells were successfully induced, mice were hydrodynamically injected with c-MYC/Mcl1 plasmids to induce HCC formation (Figure 2A). AFP immunization significantly delayed HCC tumor formation (Figure 2B, *P* < 0.0001). However, unlike Large T immunization in the Large T (+) HCC model, all immunized mice later developed tumors. *Afp* mRNA expression was similar in the eventual tumors as in control, nonimmunized, c-MYC/Mcl1 mice (Supplemental Figure 4). The data suggest that the tumors in the immunized mice were still AFP (+). However, they were able to escape from the AFP immunization, indicating that these immune-escaped tumors might be intrinsically resistant to AFP immunization.

Histological evaluation revealed that HCC lesions in control and AFP-immunized mice showed similar microscopic features, including positive expression of ectopically injected c-MYC (Figure 2C). Next, we analyzed CD8^+^ T cells in the HCC lesions and found increased CD8^+^ T cell infiltration in HCC tumors in AFP-immunized mice compared with control mice (*P* < 0.0001; Figure 2, C–F). This phenotype was most prominent in the small nodules in early stage HCC lesions (Figure 2C bottom panel, Supplemental Figure 5, A and B). To further analyze the possible immune components or pathways involved in response to AFP vaccination, we performed whole-exome RNA-Seq with samples from these 2 groups (Figure 2B and Supplemental Figure 6, A–C). GO pathway analysis demonstrated multiple significantly upregulated immune activation pathways in the AFP immunized group (Supplemental Figure 6D). Furthermore, in silico cell deconvolution using the ImmuCC algorithm (17) predicted a strong trend toward increased CD8^+^ T cells in tumors from the AFP immunized mice (Supplemental Figure 6, E and F), in line with the flow cytometry and IHC data (Supplemental Figure 1 and Figure 2, D–F).

**Figure 3:**
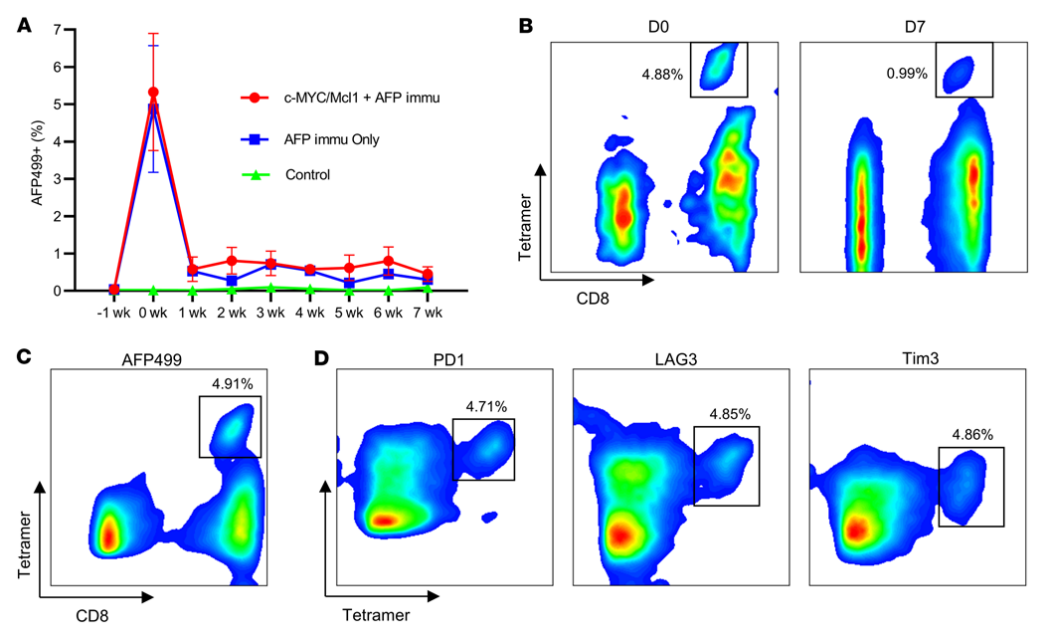
Coexpression of exhaustion markers PD1, LAG3, and Tim3 cooperates for the rapid decrease of AFP499^+^ CTLs. (**A**) AFP499^+^ CTLs decreased rapidly in the AFP immunization only or combined with the c-MYC/Mcl1 hydrodynamically injected mice. (**B**) Representative results of AFP499**^+^** CTLs at their peak (D0) or 1 week later (D7) in an AFP-immunized mouse. (**C** and **D**) The AFP499**^+^** CTLs (**C**) are observed to concomitantly express exhaustion markers, including PD1, LAG3, and Tim3. **C** and **D** are gated on the same population of CD3^+^CD8^+^ T cells. W, week(s); D, day(s); AFP499^+^, AFP499 peptidespecific CD8^+^ T cells; PD1, programmed cell death protein 1; LAG3, lymphocyte-activation gene 3; Tim3, T cell immunoglobulin and mucin protein 3; Immu, immunization.

Since AFP immunization delayed HCC development, we monitored the dynamic changes of AFP499^+^ CTLs in the peripheral blood of the immunized mice. The number of AFP499^+^ CTLs decreased rapidly after peptide induction (Figure 3A), especially after the first week (Figure 3, A and B). Subsequently, the levels of AFP499^+^ CTLs remained relatively stable, although lower. These cells could be detected 20 weeks after their first induction (Supplemental Figure 7). In addition, these T cells could be readily stimulated with a repeat dose of peptide boost, indicating they retained cell memory (Supplemental Figure 7). The same temporal pattern of CD8^+^ T cells was observed in the Large T immunized mice (Supplemental Figure 8A), suggesting a common phenotype independent of vaccine antigen.

AFP499^+^ CTLs decreased rapidly, and AFP was only expressed in c-MYC/Mcl1-induced HCC lesions several weeks later. Thus, we asked whether AFP expression at the time of AFP immunization might increase or sustain AFP499^+^ CTLs. To test this hypothesis, we coexpressed mouse AFP with c-MYC/Mcl1 plasmids 1 week after the AFP499 peptide booster in mice (c-MYC/Mcl1/AFP; Supplemental Figure 9A). Indeed, this strategy induced more AFP499^+^ CTLs 1 week after HDTVi compared with c-MYC/Mcl1 group. However, AFP499^+^ CTLs dropped and remained at similarly low levels in c-MYC/Mcl1/AFP and c-MYC/Mcl1 mice as early as 2 weeks after HDTVi (Supplemental Figure 9, B–D). AFP immunization delayed c-MYC/Mcl1/AFP-induced HCC development, although all mice developed HCC eventually, similar to the c-MYC/Mcl1 model (Supplemental Figure 9, E and F). The results suggest that AFP’s early or consistent expression did not increase AFP499^+^ CTLs or antitumor activities.

Due to the immune-escape phenotype, we hypothesized that AFP499^+^ CTLs might not be fully functional in vivo, likely due to the expression of immune exhaustion markers. Indeed, in noninjected WT mice, among all time points that we analyzed, these AFP499^+^ CTLs simultaneously expressed the PD1, LAG3, and Tim3 exhaustion markers (Figure 3, C and D). AFP499^+^ CTLs did not express CTLA4, and CLTA4 was present on CD8^–^ T cells (CD4^+^ T cells) (Supplemental Figure 8, B–D). Consistently, the coexpression of exhaustion markers of PD1, LAG3, and Tim3 was also observed on AFP499^+^ CTLs at the end-stage c-MYC/Mcl1 tumors (Supplemental Figure 10). We also performed a detailed investigation of the dynamic changes of these exhaustion markers on CD8^+^ T cells’ response to tumor progression (Supplemental Figure 11A). Interestingly, PD1 briefly increased at the point of maximal AFP-specific T cell activation (Supplemental Figure 11B). Subsequently, PD1 fell and remained low during the latent period, when tumors were controlled for 5 weeks (Supplemental Figure 11, C and D). When tumor formation and progression caused end-stage abdominal distension in approximately 7–8 weeks, this condition coincided with the upregulation of PD1, Tim3, and LAG3 exhaustion markers (Supplemental Figure 11E).

Overall, the data demonstrate that AFP immunization delayed, but could not completely prevent, c-MYC/Mcl1-driven HCC initiation in vivo. Importantly, AFP499^+^ CTLs expressed multiple immune exhaustion markers, likely suppressing the activities of AFP499^+^ CTLs and ultimately leading to immune escape.

### AFP immunization has no therapeutic efficacy against existing c-MYC/Mcl1 tumors.

**Figure 4:**
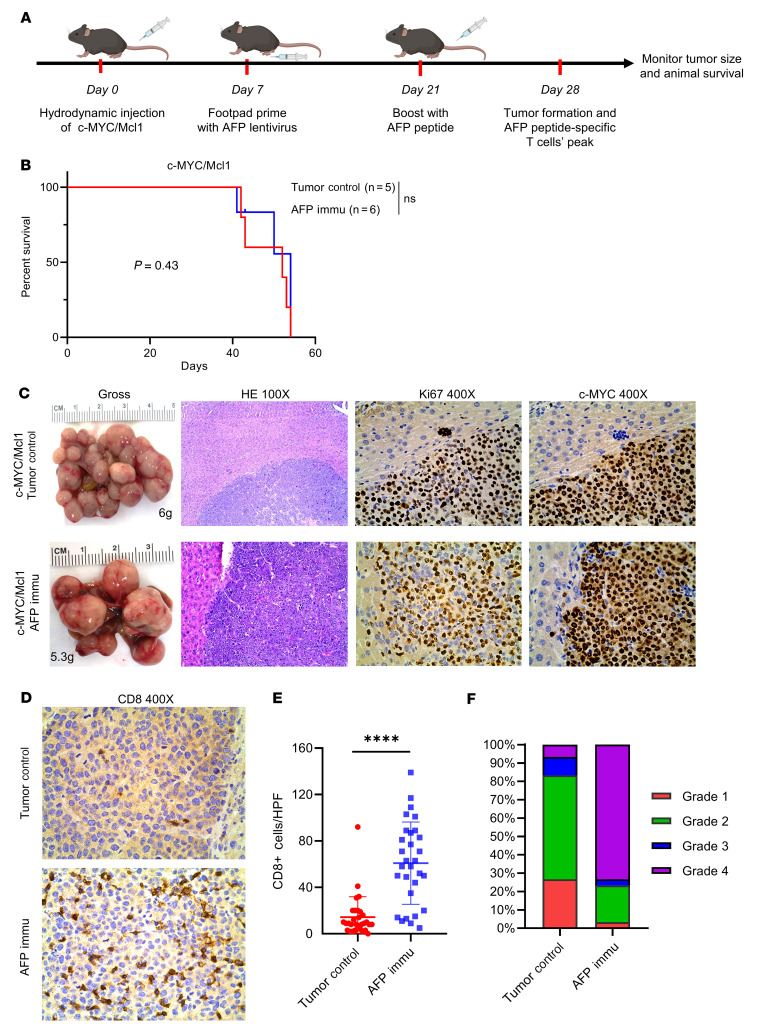
AFP immunization has no therapeutic benefits to already-existing HCC tumors. (**A**) Study design. (**B**) Survival curve of the control and AFP Immunization group mouse models. (**C**) Representative gross image and histopathological features of HE, Ki67, and c-MYC staining in the tumor control and AFP immunized group. The numbers indicate the liver weight of that particular mouse. (**D**) Representative CD8 staining of the tumor control and AFP Immunization group. (**E** and **F**) The infiltrated CD8^+^ T cells were quantified (**E**, *n* = 30 high power fields) and then graded from 1–4 based on the defined criteria; Grade 1: 0–5 cells; Grade 2: 6–20 cells; Grade 3: 21–40 cells; Grade 4: > 40 cells (**F**). Kaplan-Meier test was used for survival analysis in **B**, unpaired 2-tailed Student’s *t* test was used in (**E**). Data are presented as mean ± SD. Original magnification: 100 × and 400 ×, as indicated in column heads. HPF, high power field; Immu, immunization. ****P < 0.0001.

In our initial experiments assessing the ability of AFP vaccination to prevent HCC development, AFP499^+^ CTLs were induced before HCC initiation. To illustrate the therapeutic potential of AFP immunization for the treatment of HCC, we changed our model to generate AFP499^+^ CTLs after tumors were already developed. In the c-MYC/Mcl1 model, tumor nodules emerged from the liver around 3–4 weeks after HDTVi (Supplemental Figure 12). To evaluate the therapeutic potential of the AFP immunization strategy for preexisting tumors, mice were immunized 1 week after c-MYC/Mcl1 HDTVi (Figure 4A). Four weeks after the plasmid injection, tumor nodules began to emerge on the liver. Based on our previous studies, we would predict that at this time point, the AFP499^+^ CTLs were also present at stable levels in the mice (Figure 3, A and B). Unlike the prevention study, AFP immunization had no efficacy against existing c-MYC/Mcl1 HCC lesions. In particular, the control and experimental cohorts of mice displayed an equivalent survival length (Figure 4B, *P* = 0.43). Additional analyses showed that the tumors in these 2 groups had similar histologic features: c-MYC oncogene expression and the proliferation marker Ki67 (Figure 4C). Interestingly, the AFP-immunized tumors demonstrated abundant CD8^+^ T cell infiltration (Figure 4, D–F), suggesting that they were not functional against tumor lesions. Altogether, the data indicate that AFP immunization alone cannot inhibit the progression of preexisting HCC lesions.

### Combined AFP immunization and anti-PD1 treatment significantly suppress c-MYC/Mcl1 tumor progression.

**Figure 5:**
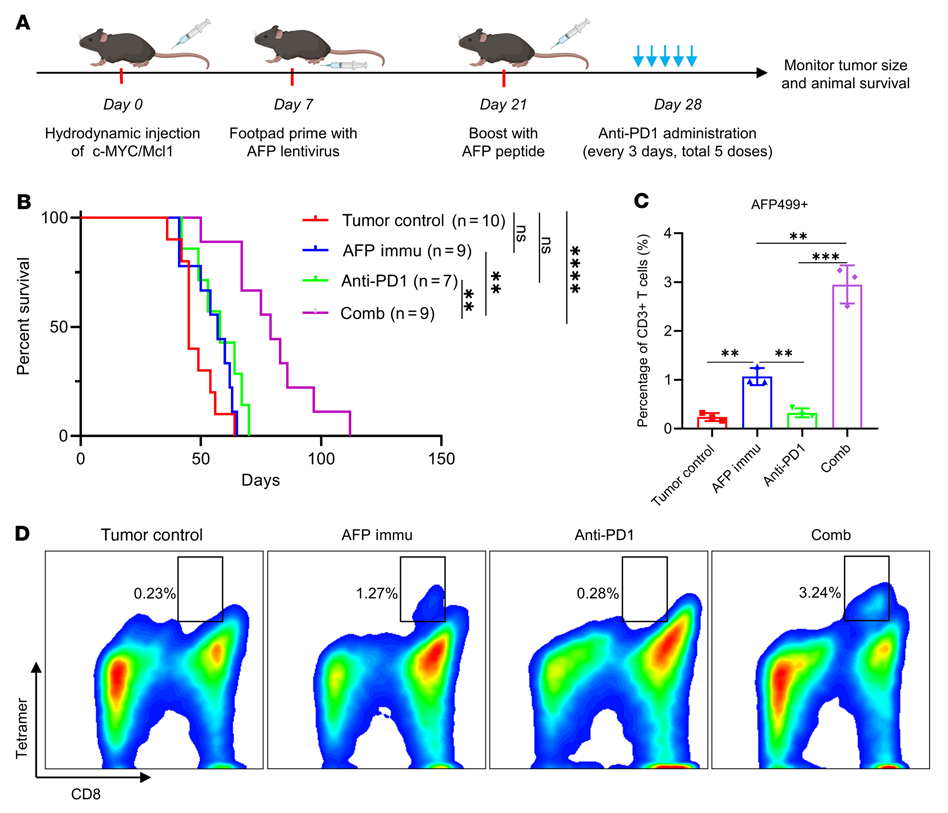
AFP immunization synergizes with anti-PD1 to inhibit c-MYC/Mcl1–induced HCC development. (**A**) Study design. (**B**) Survival curve of the various groups tested. (**C** and **D**) Quantification of the AFP499^+^ CTLs and their corresponding representative flow cytometry results in each group. Kaplan-Meier test was used for survival analysis in (**B**), 1-way ANOVA analysis was used in **C**. Data are presented as mean ± SD. **P < 0.01, ***P < 0.001. AFP499^+^, AFP499 peptidespecific CD8^+^ T cells; Comb, combination therapy; Immu, immunization.

AFP499^+^ CTLs expressed multiple exhaustion markers, including PD1 (Figure 3D). Therefore, we hypothesized that these molecules inhibited the activity of AFP499^+^CTLs and that combined AFP immunization with ICIs could hamper HCC progression. Consequently, we investigated the antitumor effect of AFP immunization combined with an anti-PD1 antibody. Treatment with 5 doses of anti-PD1 started on day 28 (and treatment was given every 3 days thereafter) after c-MYC/Mcl1 HDTVi (Figure 5A). The anti-PD1 antibody monotherapy had no efficacy in this murine HCC model, consistent with previous studies (18). In contrast, combined AFP immunization and anti-PD1 treatment significantly prolonged mouse survival (Figure 5B and Supplemental Figure 13A). Furthermore, detailed time-course studies revealed that the combination therapy considerably delayed the progression of c-MYC/Mcl1 tumor lesions (Supplemental Figure 13, B–D). Nevertheless, all mice eventually developed a high liver tumor burden and required euthanasia (Figure 5B and Supplemental Figure 13A).

Mechanistically, the combination therapy profoundly increased the percentage of AFP499^+^ CTLs in tumors compared with the AFP immunization–only group (Figure 5, C and D). IFN-γ secretion was similar in these 2 cohorts of mice (Supplemental Figure 14, A and B). There was no difference in overall CD4^+^ and CD8^+^ T cell subpopulations and the PD1 expression on CD8^+^ T cells of the tumor-infiltrating lymphocytes (TILs) between groups (Supplemental Figure 14, C–E).

In summary, the present data demonstrate that combining AFP immunization with anti-PD1 administration increases AFP499^+^ CTLs in tumor lesions, leading to delayed HCC progression. However, the overall efficacy of this combination therapy is relatively modest.

### AFP immunization synergizes with anti–PD-L1 to dramatically inhibit c-MYC/Mcl1 tumor progression.

**Figure 6:**
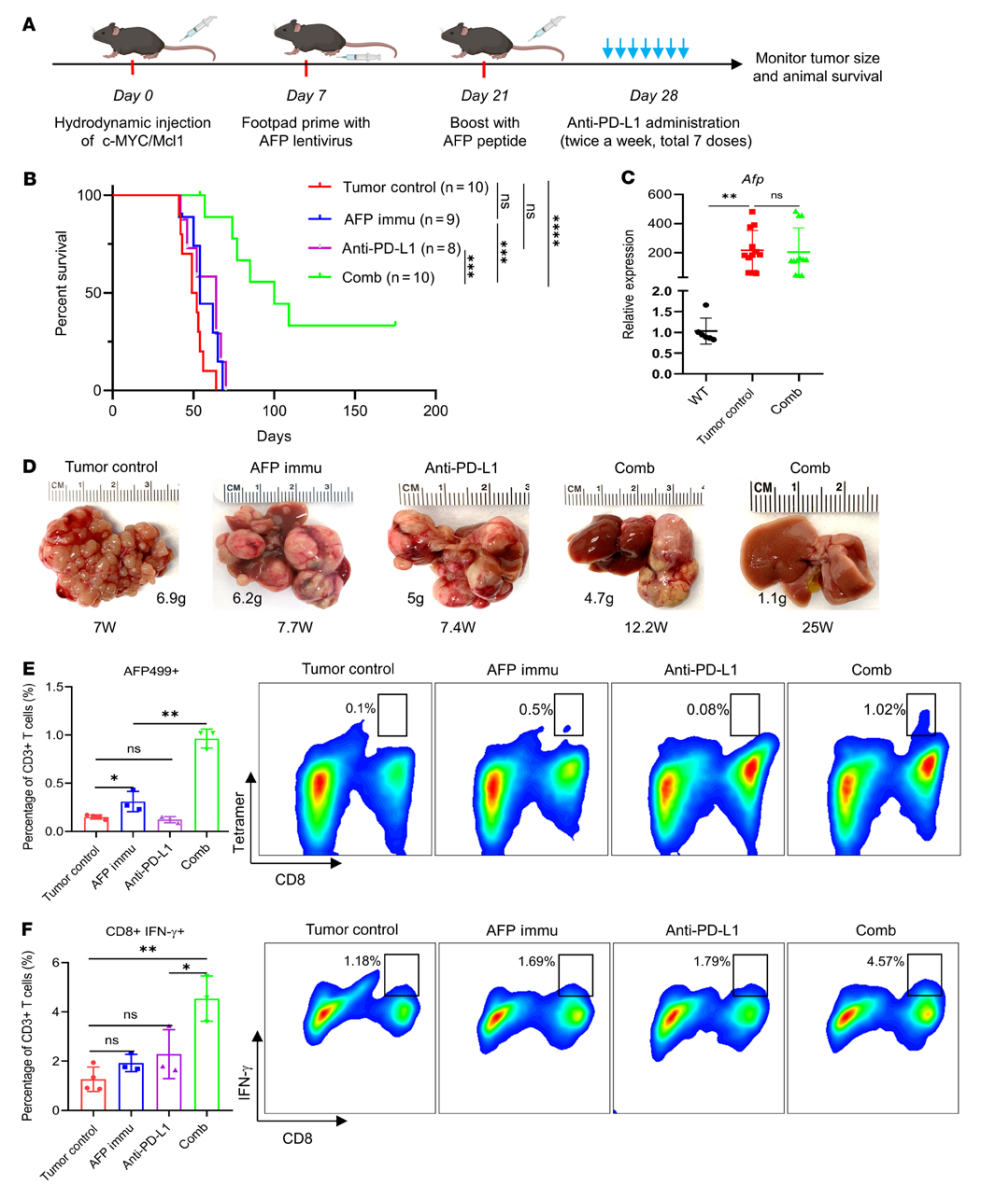
AFP immunization synergizes with anti–PD-L1 to inhibit c-MYC/Mcl1–induced HCC progression. (**A**) Study design. (**B**) Survival curve of the various groups tested. (**C**) qPCR quantification of *Afp* expression in the tumor control and combination therapy group in **B**; WT is used as normal control. (**D**) Pictures of representative livers from **B**, the numbers indicate the liver weight and weeks from injection to sacrifice date for that particular mouse. (**E**) Quantification of the AFP499^+^ CTLs and their corresponding representative flow cytometry results in each group. (**F**) Quantification of the CD8^+^IFN-γ^+^ T cells and the corresponding representative flow cytometry results in each group. Kaplan-Meier test was used for survival analysis in **B**, 1-way ANOVA analysis was used in (**C**, **E**, and **F**). Data are presented as mean ± SD. *P < 0.05, **P < 0.01. W, week(s); AFP499+, AFP499 peptide-specific CD8+ T cells; Comb, combination therapy; Immu, immunization.

We also investigated whether AFP immunization combined with anti–PD-L1 could be therapeutically effective against c-MYC/Mcl1 HCC. Treatment with 7 doses of anti–PD-L1 started on day 28 after c-MYC/Mcl1 HDTVi (Figure 6A). Consistent with a previous report, anti–PD-L1 monotherapy demonstrated no survival benefit in the c-MYC-induced HCC tumor model (19). In contrast, anti–PD-L1 administration and AFP immunization achieved a dramatic antitumor efficacy, significantly improving mouse survival (Figure 6B, *P* < 0.0001). Numerous liver tumor lesions developed in tumor control, AFP immunization, and anti–PD-L1 monotherapy mice. In contrast, only a few liver tumor lesions formed in the combination therapy group. Notably, 3 of 10 mice in the combination therapy group were tumor-free after 25 weeks of HDTVi (Figure 6, B and D, and Supplemental Table 1). The results imply that most HCC lesions disappeared upon combination therapy, with a few tumors escaping the immunotherapy. Furthermore, in the HCC lesions that developed in the combination cohort, high *Afp* mRNA expression was present (Figure 6C). This observation indicates that the tumors growing despite the combination therapy did not achieve immune escape via the downregulation of *Afp* transcription.

We performed detailed time course studies to further illustrate the antitumor activities of AFP immunization and anti–PD-L1 combination therapies (Supplemental Figure 15). While scattered tumor nodules in the liver could be seen in all mice collected 4 weeks after injection (wpi) (before treatment) (Supplemental Figure 12), multiple and larger lesions occurred in the tumor control 1 week after treatment (namely 5 wpi) (Supplemental Figure 15). In contrast, these lesions did not progress in the combination treatment cohort of mice (Supplemental Figure 15), with only some small lesions appreciable in the mouse liver. In the control group of mice, HCC continued to progress and eventually occupied the entire liver 2–3 weeks after treatment (Supplemental Figure 15). Three weeks after treatment (7 wpi), only limited HCC lesions could be found in the combination treatment mice, implying the disappearance of most tumor lesions following the combination treatment. Tumors in the various groups were histologically indistinguishable and consisted of densely packed, small basophilic-staining tumor cells, as previously reported for c-MYC/Mcl1 mice (Supplemental Figure 16, upper panel) (20). At the cellular level, elevated proliferation, assessed by Ki67 staining, was detected in early- and late-stage c-MYC/Mcl1 untreated tumors and early-stage c-MYC/Mcl1 tumors treated with the combination therapy. In contrast, proliferation was significantly reduced in the residual late-stage c-MYC/Mcl1 lesions subjected to the combination treatment (Supplemental Figure 16, lower panel). Surprisingly, similar levels of apoptosis, determined by cleaved Caspase 3 and cleaved PARP staining, occurred in the early stage of treated and untreated c-MYC/Mcl1 HCC cells. Consistent with our previous report, high levels of apoptosis characterized the late-stage c-MYC/Mcl1 untreated tumors. In contrast, few apoptotic cells were appreciable in the few residual HCC lesions from c-MYC/Mcl1 mice treated for 3 weeks with the combination therapy (Supplemental Figure 16, middle panel). These findings identify reduced proliferation but not apoptosis as the primary mechanism responsible for tumor inhibition in mice subjected to AFP immunization and anti–PD-L1 combination.

Mechanistically, AFP immunization combined with anti–PD-L1 profoundly increased the number of AFP499^+^ CTLs infiltrated in HCC tumors. Importantly, these cells were highly active as they secreted IFN-γ (Figure 6, E and F, Supplemental Figure 17, A and B, and Supplemental Figure 18E). Additionally, the combination group had more AFP499^+^ CTLs in splenocytes than controls (Supplemental Figure 18D). Furthermore, the expression of PD1 on CD8^+^ T cells was also significantly augmented in TILs and splenocytes compared with the control group, suggesting that these T cells had been highly activated (Supplemental Figure 17C and Supplemental Figure 18C).

To dissect changes in the intrahepatic immune microenvironment during the various treatments, we repeated the AFP vaccine and anti–PD-L1 treatment but terminated the experiment after administering only 2 doses of anti–PD-L1. Immune cell profiling at this time point using multiplex flow cytometry revealed a significant upregulation of CD8^+^ T cells and a downregulation of B cells in the liver tissues from the anti–PD-L1 combination group compared with the AFP immunized group and tumor control group. However, this phenotype was not observed in the spleen (Supplemental Figure 19). Notably, myeloid cells, including myeloid-derived suppressor cells (MDSCs), macrophages, and DCs, only accounted for a small proportion of immune cells both in the liver and spleen, and no difference was observed in these subpopulations (Supplemental Figure 19, C and D). The phenotype of CD8^+^ T cells was also measured. It was found that CD103^+^ liver-resident CD8^+^ T cells decreased with the combination therapy (Supplemental Figure 20, A–C), as expected from the influx of circulating T cells that became activated due to AFP immunization. In addition, the exhaustion markers PD1 and Tim3 were also high in the combination therapy (Supplemental Figure 20D). Also, CD8^+^ T cells in the liver displayed a more activated and less naive phenotype with combination therapy, as measured by CD44/CD62L staining (Supplemental Figure 20E). In summary, the findings indicate that combined anti–PD-L1 and AFP immunization increased activated CD8^+^ T cells, including AFP499^+^ CTLs, inducing the dramatic inhibition of most tumor lesions in c-MYC/Mcl1 mice.

### HCC intrinsic PD-L1 expression as a primary target of the combination therapy.

**Figure 7:**
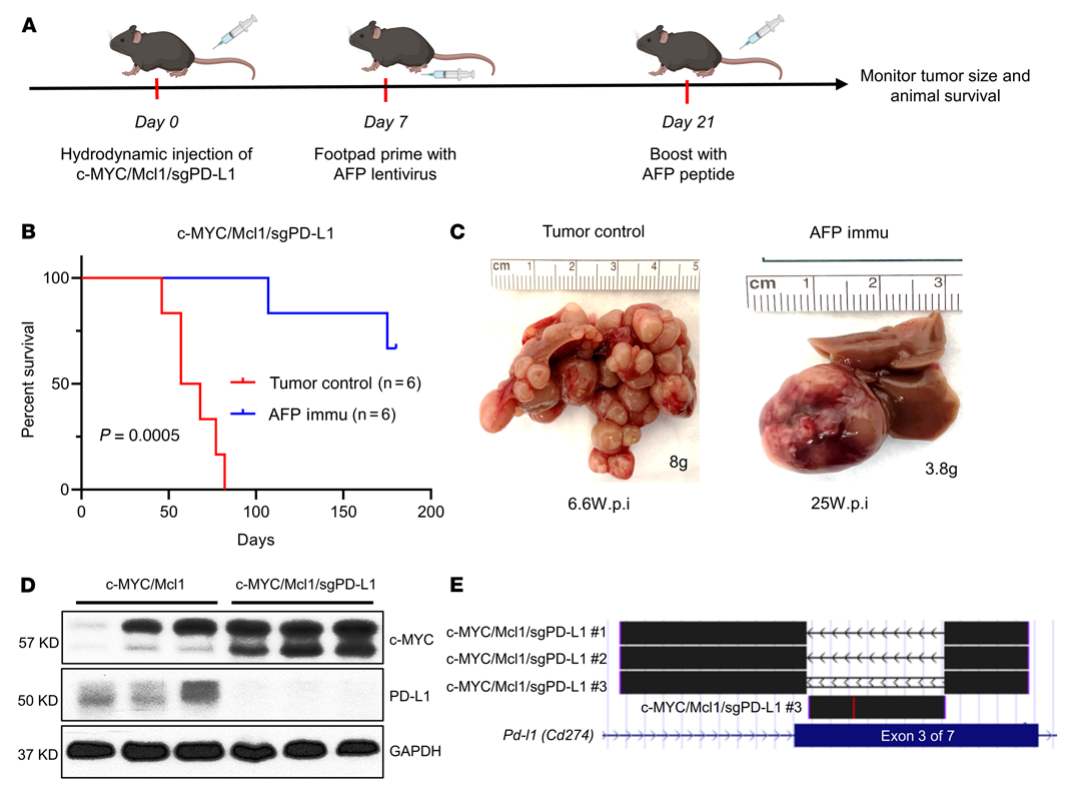
Deletion of PD-L1 does not affect tumor growth. (**A**) Study design. (**B**) Survival curve of c-MYC/Mcl1/sgPD-L1 with or without AFP immunization. Kaplan-Meier test was used for survival analysis. (**C**) Pictures of representative livers from **B**, the numbers indicate the liver weight and weeks from hydrodynamic injection to sacrifice date for that particular mouse. (**D** and **E**) Western blot (**D**) and TA cloning sequencing results (**E**) from the c-MYC/Mcl1/sgPD-L1 tumors also confirm the deletion of PD-L1 in the tumors. The TA cloning sequencing reads are aligned with mouse GRCm38/mm10 genome at the UCSC genome browser (https://genome.ucsc.edu/). PD-L1 is also called CD274, and the gRNAs are designed to target the third exon of *Pd-l1*. c-MYC/Mcl1/sgPD-L1 no. 1 and c-MYC/Mcl1/sgPD-L1 no. 2 indicate the depletion of the whole sequence between the 2 sgPD-L1 gRNAs, c-MYC/Mcl1/sgPD-L1 no. 3 indicate the reversion sequence between the 2 sgPD-L1 gRNAs. All these sequence edits cause PD-L1 gene sequence frame-shift and early stop. wpi, weeks after injection; Immu, immunization.

It has been reported that c-MYC tumors display a high expression of PD-L1 (14, 21, 22). Accordingly, PD-L1 expression was detectable in c-MYC/Mcl1 mouse HCC samples (Figure 7D). Various cells within the tumors, including HCC cells, endothelial cells, or immune cells, express PD-L1 (19). Unfortunately, IHC staining of PD-L1 was inconclusive. We therefore, investigated the PD-L1 expression in tumors using other approaches. First, we found that PD-L1 was expressed in c-MYC-derived HCC cells (Supplemental Figure 21A). Next, the profile of myeloid cells and the expression of PD-L1 in the tumor immune microenvironment were investigated by flow cytometry. There were no differences in the proportion of the respective myeloid subtype cells within treatment groups (Supplemental Figure 22, A and B). MDSC subsets displayed the most abundant PD-L1 expression in both tumors with or without AFP immunization, consistent with their known immunosuppressive role (Supplemental Figure 22, C and D). Macrophages and DCs exhibited relatively lower expression of PD-L1. These data suggest that PD-L1 is expressed in HCC cells and the immune cells in tumor lesions.

Next, we asked whether HCC-intrinsic expression of PD-L1 may be one of the targets of anti–PD-L1 treatment. Toward this goal, we applied CRISPR-Cas9–based gene editing, deleted *Pd-l1* in tumor cells, and tested whether ablation of PD-L1 in HCC cells recapitulated anti–PD-L1 activities during the combination therapy. As the first step, we deleted PD-L1 in mouse HCC cell lines. The study proved the effectiveness of deleting *Pd-l1* in HCC cells, and deletion of *Pd-l1* did not affect c-MYC HCC cell proliferation in vitro (Supplemental Figure 21, B–D). Next, we coinjected c-MYC/Mcl1 and sgPD-L1 plasmids into the mice. This strategy allowed the deletion of *Pd-l1* specifically in the mouse hepatocytes while coexpressing c-MYC/Mcl1 oncogenes. Notably, hepatocyte-specific ablation of PD-L1 did not affect c-MYC/Mcl1-induced HCC development (Figure 7, A and B). The results were consistent with the lack of therapeutic efficacy of the anti–PD-L1 antibody as monotherapy. Next, we treated the c-MYC/Mcl1/sgPD-L1 mice with the AFP vaccine (Figure 7A). The results showed that *Pd-l1* deletion in c-MYC/Mcl1 tumors plus AFP immunization significantly prolonged mouse survival and dramatically reduced the number of liver tumor nodules (Figure 7, B and C), recapitulating what was observed in AFP immunization and anti–PD-L1 combination therapy (Figure 6). Western blotting and sequencing of the tumor cells confirmed the successful deletion of PD-L1 in the eventual HCC samples (Figure 7, D and E).

The data demonstrate PD-L1 expression in both HCC and immune cells within the tumors. HCC cell–intrinsic PD-L1 was one of the primary targets of the anti–PD-L1 antibody during the combination therapy. However, at this stage, we cannot exclude the possible involvement of PD-L1 in immune cells in the tumor-suppressing activities of the anti–PD-L1 antibody.

### AFP immunization synergizes with anti–PD-L1 to inhibit cMet/β-catenin tumor progression.

Since more than half of patients with HCC display elevated AFP expression, we aimed to expand the potential application of the AFP vaccine combination immunotherapy strategy for AFP(+) HCC driven by other oncogenes. Thus, we expanded the investigation to the well-established cMet/β-catenin mouse HCC model, which displays immune-escape features and resistance to conventional immunotherapy (23). Furthermore, the cMet/β-catenin HCC model exhibits a similar AFP expression to the c-MYC/Mcl1 model (Supplemental Figure 23B).

Consistent with the c-MYC/Mcl1 prevention model (Figure 2, A and B), the AFP immunization also prevented cMet/β-catenin-induced HCC tumor formation (Supplemental Figure 23, A C, and D, *P* < 0.05). The immune escape profile of the cMet/β-catenin–induced HCC model was dissected to develop a potential AFP immunization combination therapy. The macrophages were significantly decreased, and the macrophage expression of PD-L1 was upregulated (Supplemental Figure 24, B and D). Both polymorphonuclear-MDSCs (PMN-MDSC) and monocytic-MDSCs (M-MDSC) expressed the highest baseline PD-L1 levels (Supplemental Figure 24, C and D), which was consistent with the profile of c-MYC/Mcl1 tumors (Supplemental Figure 22).

**Figure 8:**
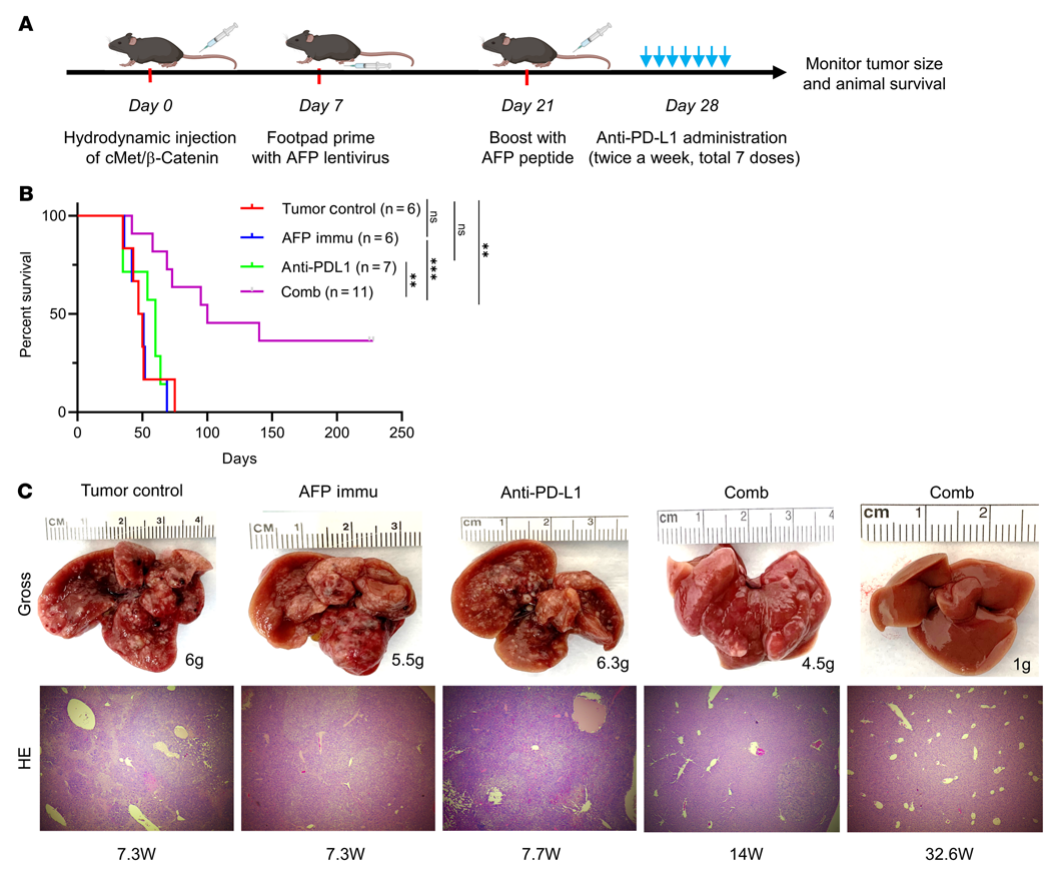
AFP immunization synergizes with anti–PD-L1 administration to inhibit cMet/β-catenin–induced HCC progression. (**A**) Study design. (**B**) Survival curve of the various groups tested. (**C**) Representative liver gross images and HE results (original magnification: 100 ×) of the investigated groups from **B**, the numbers indicate the liver weight and weeks from injection to sacrifice date for that particular mouse. Kaplan-Meier test was used for survival analysis in **B**. **P < 0.01, ***P < 0.001. W, week(s); Comb, combination therapy; Immu, immunization.

To further illustrate the therapeutic potential of the AFP immunization combination strategy in the cMet/β-catenin model, mice were AFP immunized 1 week after HDTVi (Figure 8A). As expected, anti–PD-L1 monotherapy or AFP immunization demonstrated no survival benefit in the already formed cMet/β-catenin HCC tumors. However, the combination therapy of AFP vaccination and anti–PD-L1 treatment showed a remarkable antitumor effect in this model (Figure 8, B and C). These results suggest that combination therapy might be an effective strategy for AFP(+) HCC regardless of the oncogenic signature.

## Discussion

While targeting AFP for HCC immunotherapy is a promising therapeutic approach for treating AFP(+) HCC, limited clinical achievements have been reported to date. In particular, few preclinical studies have evaluated the efficacy of the AFP vaccine in animal models, which are critical to providing guidance and support for further clinical investigations. The engineered T cells conferring the AFP epitope–specific T cell receptor (TCR-T) or chimeric antigen receptor (CAR-T) is another strategy for HCC treatment (7, 8, 24–26). However, the severe off-target toxicity of the engineered T cells is a primary concern of its application in the clinic, suggesting that safety and efficacy must be considered in targeting AFP-based HCC immunotherapy (9, 27–29). In the current study, we performed a detailed preclinical investigation to evaluate AFP immunization in preventing c-MYC/Mcl1 HCC initiation and inhibiting HCC progression. This approach effectively delayed HCC formation in mice, suggesting that the AFP-based vaccine may be helpful as an immunoprevention strategy against HCC in humans. Unlike other cancer types, HCC has a well-defined etiology, including HBV or HCV chronic infection and NAFLD/NASH. Therefore, one can envision that AFP-based vaccines might be helpful in preventing or significantly delaying the onset of HCC in this population, particularly at-risk patients who have cirrhosis and are enrolled in a radiographic screening program. Further clinical studies are necessary to develop effective and safe humanized vaccines against AFP. In addition, as not all HCCs are AFP(+), it is important to identify additional tumor antigens to target for immunization for cancer prevention studies.

Another significant result from our preclinical study is that AFP immunization has no efficacy against already-formed c-MYC/Mcl1 tumor lesions due partly to immune escape from AFP-specific T cells. Similar results were also obtained in the cMet/β-catenin model. It is worth noting that we only tested AFP immunization in these 2 murine HCC models in the current investigation. Therefore, it is crucial to expand the study to additional preclinical HCC models, either oncogene-driven or chemically induced. Nevertheless, the present data have relevant clinical implications, suggesting that AFP immunization might have limited value as monotherapy for established HCC, as shown by previous clinical trials (6–8). Instead, AFP immunization should be applied in association with additional treatments as combination therapies that address the underlying resistance mechanisms.

We tested the therapeutic potential of AFP immunization and anti-PD1 or anti–PD-L1 antibodies in c-MYC/Mcl1 mice. While both combination treatments could suppress HCC progression, leading to prolonged mouse survival, distinct phenotypes were observed. Indeed, the AFP immunization/anti-PD1 combination therapy delayed HCC progression, whereas the AFP immunization/anti–PD-L1 combination therapy strongly inhibited the development and progression of most HCC nodules (Supplemental Figure 25). In addition, both combination therapies increased the presence of AFP499^+^ CTLs in the TILs. However, only the AFP immunization/anti–PD-L1 combination increased functional AFP499^+^ CTLs. Furthermore, we analyzed AFP499^+^ CTLs in AFP-immunized WT mice dosed with anti-PD1 and anti–PD-L1 antibodies, respectively (Supplemental Figure 26A). Our preliminary data showed that anti–PD-L1, but not anti-PD1 antibody, slowed the decrease of the AFP499^+^ CTLs (Supplemental Figure 26B). Thus, these findings indicate that AFP immunization/anti–PD-L1 combination treatment, but not AFP immunization/anti-PD1 combination, might prolong mouse survival and enhance the function of these AFP499^+^ CTLs in vivo. The few remaining tumor nodules in the livers of the AFP immunization with the anti–PD-L1 combination group may be due to the highly suppressive tumor microenvironment of HCC (30). However, additional studies are required to elucidate the molecular mechanisms of this observation further.

The expression of PD-L1 correlates strongly with response to immunotherapy, but the ways of quantifying PD-L1 expression vary depending on the identity of the expressing cell (31, 32). Deleting PD-L1 in the MYC/Mcl1 model was sufficient to sensitize established tumors to AFP vaccination and represent a major source of checkpoint-based immune escape. However, PD-L1 was also found to be expressed variably in myeloid cells. Macrophages and dendritic cells are often polarized toward exerting either a pro- or antitumor function, while MDSCs, which had the highest PD-L1 expression, are generally considered protumor. In addition, eliminating macrophages and MDSCs have been performed in other tumor models of HCC that have sensitized liver tumors to immunotherapy. Thus, it is possible that, in addition to tumor cells, PD-L1 expression on myeloid cells can contribute to immune escape. This hypothesis may also explain residual tumors from immunized mice that were generated from HDTVi of c-MYC/Mcl1/sgPD-L1. Further studies on the effect of myeloid-specific PD-L1 deletion are warranted.

Another intriguing observation from the investigation is that AFP(+) HCC lesions could develop in the presence of AFP immunization and anti–PD-L1 combination therapies. To further investigate the molecular mechanisms of the immune-escaped HCC, we performed RNA-Seq analysis for normal liver tissues, c-MYC/Mcl1 HCC, AFP immunized c-MYC/Mcl1 HCC, and eventual HCC lesions from combination therapies. PCA analysis revealed that the immune-escaped HCC shared similar global gene expression profiles with control c-MYC/Mcl1 HCCs (Supplemental Figure 27A). The results were consistent with similar investigations from other groups (19, 23). Previous studies suggest that the activated Wnt/β-catenin pathway may induce an immune-escape phenotype in HCC (23). However, we found no evidence of Wnt/β-catenin pathway activation in the immune-escaped c-MYC/Mcl1 HCC lesions (Supplemental Figure 27B and Supplemental Figure 28). Nevertheless, we found that several essential genes involved in the IFN-γ signaling pathway and chemokines that could recruit circulating leukocytes to inflammatory sites might be impacted by the combination therapy (33). For instance, the combination therapy induced interferon regulatory factor 1 (IRF1), an IFN regulatory factor that could activate the transcription of proinflammatory chemokines (Supplemental Figure 27C) (34). Chemokine ligand 4 (CCL4), which plays a critical role in tumor immune cell infiltration, was also significantly induced in the combination group (Supplemental Figure 27D) (35). Whether these molecules contribute to the response to the eventual HCC inhibition in combination therapy needs further investigation. Most likely, these immune-escaped HCCs have an aberrant immune-cell microenvironment, which may not be adequately analyzed via bulk RNA-Seq studies. Instead, single-cell RNA-Seq studies may be required to obtain a comprehensive picture of the immune microenvironment of control and immune-escaped HCC lesions. Such studies may provide detailed information about the different immune cell populations in the HCC lesions and the genes expressed in diverse immune cell populations. The data would guide us to better understand the molecular mechanisms leading to the resistance of the combination immunotherapy.

## Methods

### Mouse, plasmids, and HDTVi.

C57BL/6 mice were housed and monitored at the University of California, San Francisco. The plasmid vectors used were as follows: pT3EF1α-c-MYC, pT3EF1α-Mcl1, pT3EF1α-AFP, pT3EF1α-Large T, PX330, PX330-sgPD-L1, and SB transposase. Mouse HDTVi was performed as described previously (20).

### Mouse immunization and tetramer detection.

C57BL/6 mice were footpad immunized with AFP or Large T lentivirus and boosted with AFP499 (SSYSYRRL, H-2K^b^) or Large T (SAINNYAQKL, H-2D^b^) peptide. The antigen-specific CD8^+^ T cells were detected with anti-AFP or anti–Large T tetramer antibody (NIH Tetramer Core Facility).

### Checkpoint inhibitor administration.

Anti-mouse PD-1 antibody was injected IP on days 28, 31, 34, 37, and 40 after HDTVi. In addition, the anti-mouse PD-L1 antibody was administered IV on day 28 at 10 mg/kg, followed by 5 mg/kg IP on days 31, 35, 38, 42, 45, and 49 after HDTVi.

### Statistics.

All data are presented as mean ± SD. Statistical analysis was performed using GraphPad Prism V8.0. Unpaired 2-tailed Student’s *t* test was used to compare every 2 groups, the 1-way ANOVA was used to compare more than 2 groups, and the Kaplan-Meier test was used for survival analysis. *P* < 0.05 was considered significant.

### Study approval.

The Committee for Animal Research at the University of California, San Francisco, approved all experimental procedures with animals (No. AN173073), which were also consistent with the ethical guidelines of the US NIH. Housing and maintenance were provided by the University of California, San Francisco, and all animals were fed a standard chow diet with free access to water.

## Author contributions

XL, SD, JX, CL, and XC conceived and designed the studies. XL, SD, JX, BLG, HZ, GC, Y Zhou, Y Zhang, HX, and FZ generated data, key reagents, and samples. XL, SD, JX, BLG, RM, SZ, TC, and ME analyzed and interpreted the data. XL, SD, JX, BLG, HZ, GC, Y Zhou, Y Zhang, HX, FZ, RM, SZ, TC, ME, DFC, YH, CL, and XC wrote and revised the manuscript. All authors have read and approved the manuscript. The order of the co–first authors was determined by their efforts and contributions to the manuscript.

## Supplementary Material

Supplemental data

## Version 1 04/11/2023

In-Press Preview

## Version 2 06/01/2023

Electronic publication
