# Supplementary material for: Combination of AFP vaccine and immune checkpoint inhibitors slows hepatocellular carcinoma progression in preclinical models
Source: J Clin Invest. 2023 Jun 1;133(11):e163291. doi: 10.1172/JCI163291 (PMC10231990; doi:10.1172/JCI163291)
Supplement: Supplemental data [file jci-133-163291-s058.pdf]

Combination of AFP vaccine and immune checkpoint inhibitors slows hepatocellular carcinoma progression in preclinical models

Xinjun Lu[1,#], Shanshan Deng[2,#], Jiejie Xu[3,#], Benjamin L. Green[4,#], Honghua Zhang[1], Guofei Cui[2], Yi Zhou[2,5], Yi Zhang[2,6], Hongwei Xu[2,7], Fapeng Zhang[1], Rui Mao[8], Sheng Zhong[2], Thorsten Cramer[9,10], Matthias Evert[11], Diego F. Calvisi[11], Yukai He[8], Chao Liu[1,*] and Xin Chen[2,4,*]

Table of content

Supplementary Materials and Methods……………………………….…………………………..3

Supplementary Figure 1…………………………………………….……………………………..8

Supplementary Figure 2…………………………………………….……………………………..9

Supplementary Figure 3…………………………………………………………………………..10

Supplementary Figure 4…………………………………………………………………………..11

Supplementary Figure 5…………………………………………………………………………..12

Supplementary Figure 6…………………………………………………………………….......13

Supplementary Figure 7…………………………………………………………………….......15

Supplementary Figure 8…………………………………………………………………….......16

Supplementary Figure 9…………………………………………………………………….......17

Supplementary Figure 10…………………………………………………………………........18

Supplementary Figure 11…………………………………………………………………........19

Supplementary Figure 12…………………………………………………………………........20

Supplementary Figure 13…………………………………………………………………........21

Supplementary Figure 14…………………………………………………………………........23

Supplementary Figure 15…………………………………………………………………........24

Supplementary Figure 16…………………………………………………………………........25

Supplementary Figure 17…………………………………………………………………........26

Supplementary Figure 18……………………………………………………………………......27

Supplementary Figure 19……………………………………………………………………......28

Supplementary Figure 20……………………………………………………………………......29

Supplementary Figure 21……………………………………………………………………......31

Supplementary Figure 22……………………………………………………………………......32

Supplementary Figure 23……………………………………………………………………......33

Supplementary Figure 24……………………………………………………………………......34

Supplementary Figure 25……………………………………………………………………......35

Supplementary Figure 26……………………………………………………………………......36

Supplementary Figure 27……………………………………………………………………......37

Supplementary Figure 28……………………………………………………………………......38

Supplementary Table 1……………………………………………………….……………......39

Supplementary references……………………………………………….……………......40

Supplementary Material and Methods

Mouse, plasmids, and hydrodynamic tail vein injection (HDTVi)

C57BL/6 mice were purchased from the Jackson Laboratory (Sacramento, CA, USA). Mice were housed and monitored per protocols approved by the Committee for Animal Research at the University of California, San Francisco. The plasmids vector of pT3EF1α and pLentivirus containing AFP and SV40 Large T (Large T) were generated by gateway LR reaction from pENTR1A-AFP and pENTR1A-Large T (Addgene, #22297). The pENTR1A-AFP was generated by PCR amplifying the AFP gene with mouse cDNA and then cloned into the pENTR1A vector. The gRNA sequence (GTTTACTATCACGGCTCCAA, GGGGAGAGCCTCGCTGCCAA) for PX330-sgPD-L1 was previously reported (1). The PX330 vector was from Zhang's lab (Addgene, #42230). The pT3EF1α-c-MYC, pT3EF1α-Mcl1, and pCMV-sleeping beauty transposase (SB) have been described previously (2). Briefly, 10μg of pT3EF1α-c-MYC and pT3EF1α-Mcl1, with or without 30μg of pT3EF1α-AFP, 30μg pT3EF1α-Large T, or 60μg of PX330-sgPD-L1, and SB plasmid at the 25:1 ratio to all pT3EF1α plasmids were diluted in 0.9% NaCl solution and injected in the volume corresponding to 10% of the mouse weight via tail vein in ~5 seconds. Mice were sacrificed at indicated time points or when they became moribund.

Mouse immunization and tetramer detection

Age-matched 6-8 weeks old male or female C57BL/6 mice were footpads immunized with 10^7 transduction units of AFP or Large T lentivirus and boosted with 200μg AFP499 (SSYSYRRL, H-$2K^b$) or Large T (SAINNYAQKL, H-$2D^b$) peptide, each in combination with adjuvant of 50μg anti-CD40 antibody (Bio X Cell, Lebanon, NH; clone FGK45) plus 50μg poly(I:C) (InvivoGen, San Diego, CA) 2 weeks later after tail vein injection. One week after the peptide boost, mouse peripheral blood mononuclear cells were collected to detect antigen-specific CD8+ T cells with APC conjugated anti-AFP or anti-Large T tetramer antibody for 30 minutes, followed by T cell

surface or intracellular marker staining. The NIH Tetramer Core Facility synthesized all tetramers at Emory University.

Tumor-infiltrating lymphocyte isolation

For liver tumor-infiltrating lymphocytes (TILs), the liver was perfused with 1×PBS by portal vein perfusion. Subsequently, ~1.5g liver tumors were minced to 3-5 mm^3 fragments and digested at room temperature for 1.5 hours in HBSS supplemented with 1mg/ml type IV Collagenase (Sigma-Aldrich, St. Louis, MO; C5138), 200U/ml type IV DNase (Sigma-Aldrich, D5025), 0.1mg/ml Hyaluronidase (Sigma-Aldrich, H6254). The digested suspension was passaged through a 100μm cell strainer to remove undigested fragments and fat tissue. After centrifugation, the pellet was resuspended in 40% Percoll and overlaid on top of 80% Percoll. After gradient separation, the TILs were harvested between 40% and 80% Percoll for downstream analysis. Lymphocytes from the spleen were prepared by gentle maceration through a 70μm strainer. The lymphocyte suspensions were achieved by red blood cell lysis, and then all suspensions were passed through a 40μm strainer for downstream analysis.

Flow cytometry

Fluorescently conjugated anti-mouse antibody CD3 Alexa Fluor 594 (100240), CD3 FITC (17A2), CD4 BV605 (100451), CD4 PE (GK1.5), CD8 BV605 (53-6.7), CD11b BV421 (101224), CD11b Pacific Blue (101224), CD11c BV650 (117339), CD19 APC (152410), CD44 PE/Cy7 (103030), CD62L PerCP/Cy5.5 (104432), CD103 FITC (121420), F4/80 FITC (123108), I-A/I-E (MHC class II) APC (107614), Ly-6C APC/Cy7 (128026), Ly-6G Alexa Fluor 700 (127622), NK1.1 Pe-Cy7 (108714), PD1 BV421 (29F.1A12), PD1 PE/Cy7 (109110), PD-L1 PE (124308), TCR γ/δ PE (118108), and anti-rat IgG2bκ PE (400636) were purchased from BioLegend (San Diego, CA). CD8 BV711 (563332) was purchased from BD Biosciences (San Jose, CA). In addition,

fluorescently conjugated anti-mouse CD45 Alexa Flour 700 (30-F11), CD3 PE-Cy7 (145-2C11), Tim3 PerCP-eFlour 710 (RMT3-23), LAG3 Super Bright 702 (C9B7W), IFN-γ FITC (XMG1.2), Foxp3 PE-Cy5 (FJK-16s), and CD16/32 (93) were obtained from Thermo Fisher Scientific (Waltham, MA). The viability dye Zombie Aqua (BioLegend, San Diego, CA) was applied for 20 min at 4°C. Fc receptor blocking was performed with anti-CD16/CD32. TILs and splenocytes intracellular cytokine staining was accomplished through 4 hours of stimulation with cell stimulation cocktail plus protein transport inhibitors (Thermo Fisher Scientific, Cat. 00-4975-93) *in vitro*. IFN-γ staining was achieved through fixation and permeabilization with the intracellular fixation/permeabilization kit (Thermo Fisher Scientific, Cat. 88-8824-00) according to the manufacturer's protocol. Flow cytometry was performed on a Fortessa Cytometer (BD Biosciences, San Jose, CA), and the data were analyzed with the FlowJo V10.4.2 software (Tree Star Inc).

Checkpoint inhibitor administration

For checkpoint inhibitor studies, 200μg of anti-mouse PD1 (RMP1-14; Bio X Cell) antibody was injected intraperitoneally (IP) on days 28, 31, 34, 37, and 40 post-HDTVi (every 3 days, total 5 doses). In addition, the anti-mouse PD-L1 antibody (clone 6E11) was a gift from Genentech (San Francisco, CA). It was administered intravenously on day 28 at 10mg/kg, followed by 5mg/kg IP on days 31, 35, 38, 42, 45, and 49 post-HDTVi (twice a week, total 7 doses).

Cell lines, protein extraction, and Western blot analysis

The HCC cell lines HCC3-4 and HCC4-4 were gifts from Dr. Dean Felsher at Stanford University (3). The sgPD-L1 gRNA sequence and the no target sgEGFP gRNA sequence (GGGCGAGGAGCTGTTCACCG) were cloned into the CRISPR lentivirus vector. HCC cell lines were transfected with respective CRISPR lentivirus and selected with puromycin. Proteins were

extracted from mouse liver tissue and cells as described previously (4). The antibodies used for Western blotting are as follows: c-MYC (Abcam, ab32072), PD-L1 (Abcam, ab213480), SV40 Large T (Millipore-Sigma, PAb416), GAPDH (Cell Signaling, 5174), β-Tubulin (Cell Signaling, 2128).

Histology and immunohistochemistry

Liver tissues were cut into a ~0.5 cm^3, fixed in 10% formalin, and embedded in paraffin. The hematoxylin and eosin (HE) and immunohistochemistry (IHC) stainings were performed as previously described (4). HE-stained slides were imaged by a Leica microscope, and the HE-stained tumor area was quantified by automated area counting in ImageJ. The primary antibodies used for IHC are as follows: β-Catenin (BD Biosciences, 610153), CD8 (Thermo Fisher Scientific, 4SM15), c-MYC (Abcam, ab32072), Cleaved Caspase 3 (Cell Signaling, 9661), Cleaved PARP (Cell Signaling, 94885), Glutamine Synthetase (BD Biosciences, 610517), Ki67 (Cell Signaling, 12202), PD-L1 (Cell Signaling, 64988). Slides were then incubated with secondary antibody and VECTASTAIN Elite ABC (Vector Laboratories, Burlingame, CA; PK-6100) and stained with DAB (Vector Laboratories; SK-4105).

RNA sequencing and bioinformatics data analysis

HCC tumor total RNAs were extracted with Trizol reagent, and RNA integrity was evaluated by Agilent 2100 Bioanalyzer following the manufacturer's instructions. Bulk RNA and whole-exome RNA sequencing (RNA-seq) was performed by Novogene (Sacramento, CA), and raw data were prepared as described before (5). The aligned gene read counts were used for differential expression gene analysis using the DESeq2 package V1.34.0 in R. The significantly differentially expressed genes were defined by having 1.5 fold or more changes with adjusted P values ≤0.05. The ClusterProfiler package V4.2.0 was used for characterizing functional enrichment GSEA analysis (6). Whole-exome RNA-seq data analysis, including normalization, QC, DEGs, and GO

pathway analysis, was performed on the iDEP platform(7) (v0.96, accessed 1/30/2023). In silico immune cell deconvolution was performed using ImmunCC(8) (accessed 1/30/2023)

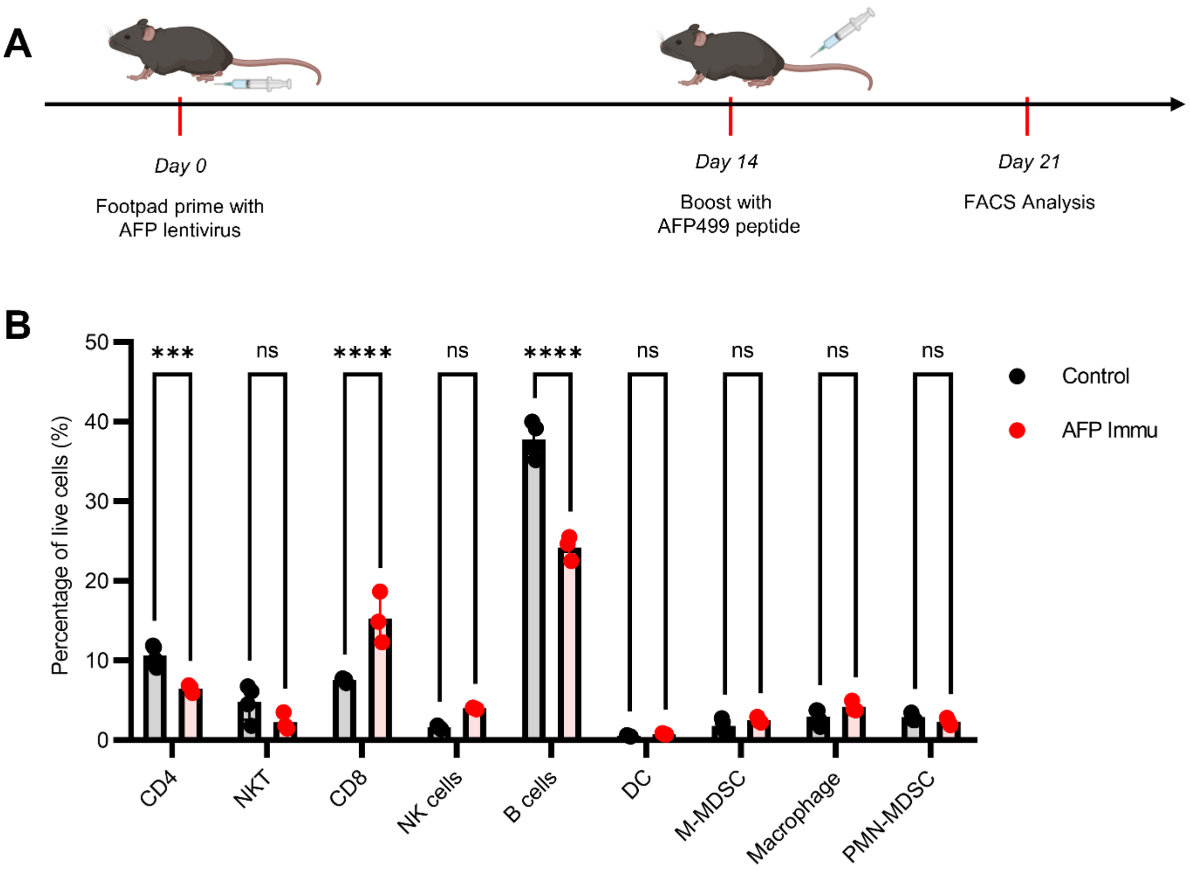

Supplementary Figure 1: Analysis of the liver immune cell landscape response to AFP immunization in tumor-free mice. (A) Tumor-free mice livers were harvested post-AFP immunization for immune cell flow cytometry analysis. (B) Quantification of the major immune cell types from mice livers with or without AFP immunization. Unpaired two-tailed student's t-test was used between respective groups in (B), ns $p > 0.05$, * $p \leq 0.05$, ** $p \leq 0.01$, *** $p \leq 0.001$, **** $p \leq 0.0001$. Abbreviations: ns, no significance; Immu, immunization; DC, dendritic cell; MDSC, myeloid-derived suppressor cell; M-MDSC, monocytic-MDSC; PMN-MDSC, polymorphonuclear-MDSC; NKT, natural killer T cell; NK, natural killer cell.

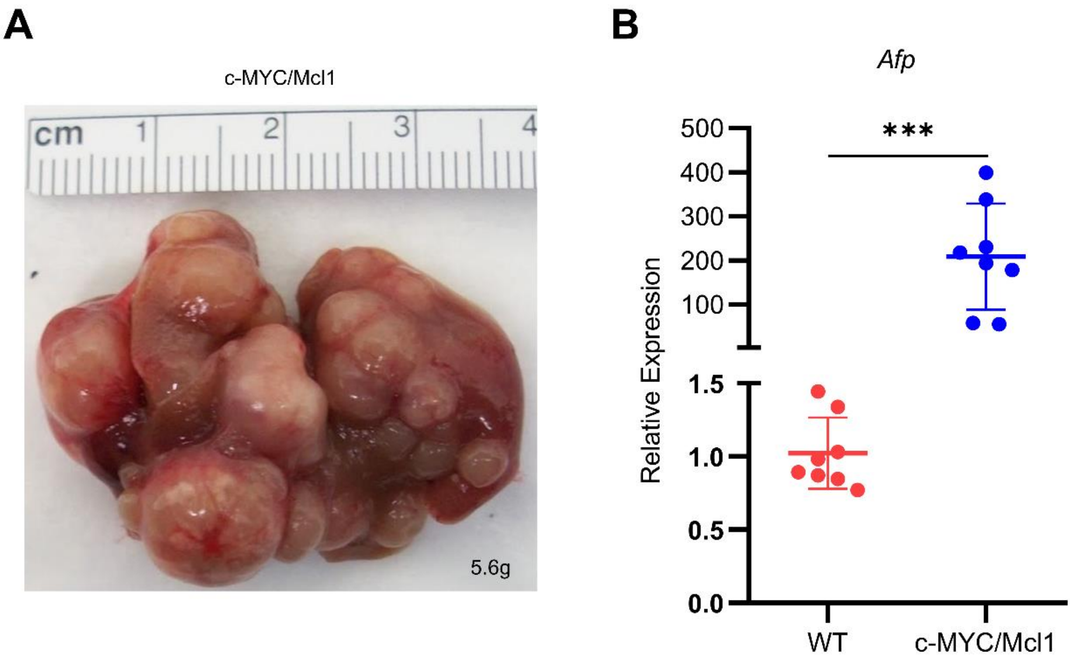

Supplementary Figure 2. AFP expression in c-MYC/Mcl1 tumors. (A) Representative liver macroscopy picture from a c-MYC/Mcl1 tumor. (B) Relative expression of *Afp* in mouse wild-type and c-MYC/Mcl1 liver tumors. Unpaired two-tailed student's t-test was used, ***P < 0.001.

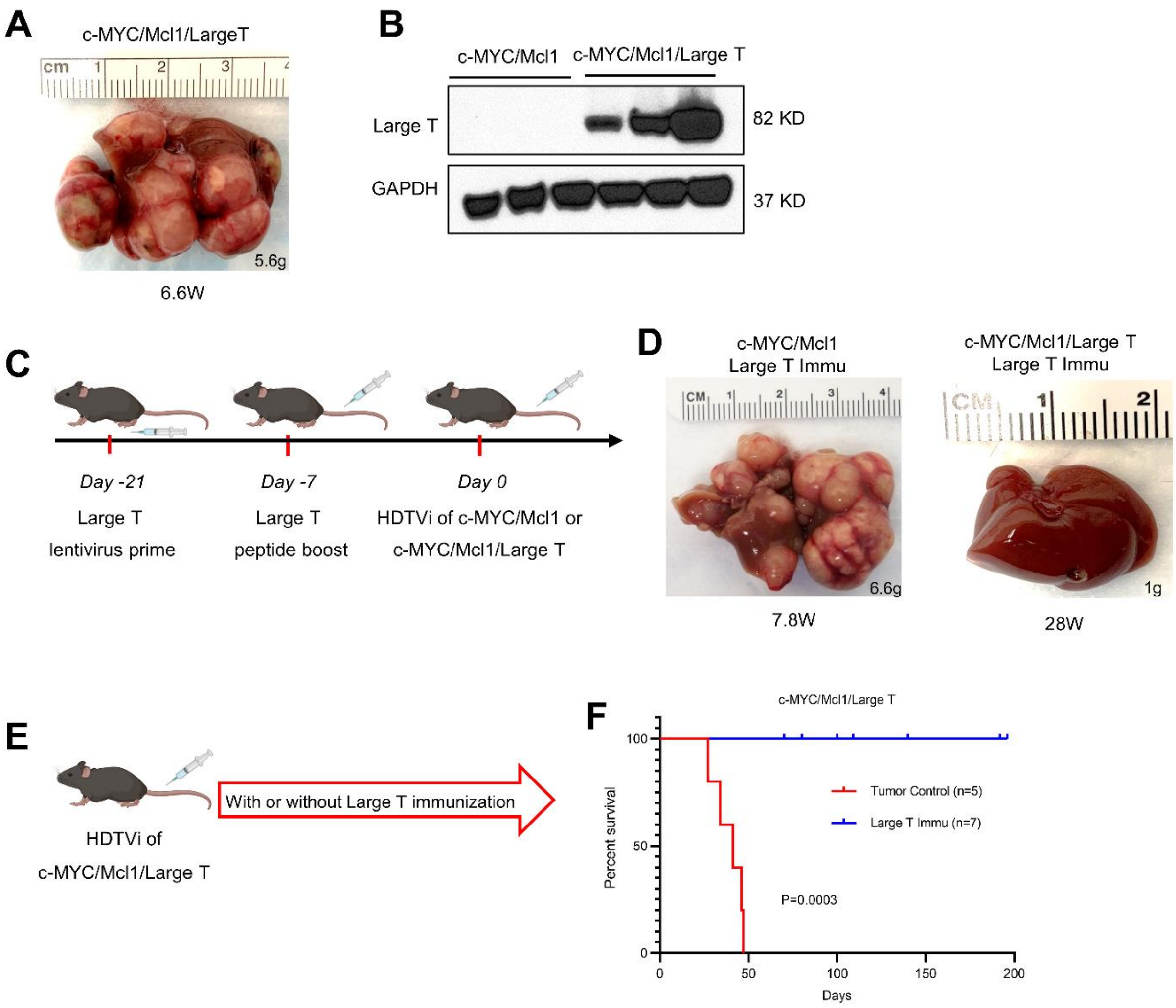

Supplementary Figure 3. Allogenic antigen Large T immunization completely eradicates c-MYC/Mcl1/Large T tumor formation. (A) Representative macroscopy picture from a c-MYC/Mcl1/Large T induced mice liver tumor, and the Western blot analysis results of the expression of Large T protein within the tumor (B). (C) Study design and representative pictures from the c-MYC/Mcl1 and c-MYC/Mcl1/Large T with Large T immunization livers (D). (E) Study design and survival curve of the tumor control group and Large T immunized group of the c-MYC/Mcl1/Large T mice (F). Kaplan-Meier test was used for survival analysis in (F). The numbers indicate the liver weight and weeks from plasmids injection to the sacrifice date for that particular mouse. Abbreviations: W, week(s); Immu, immunization.

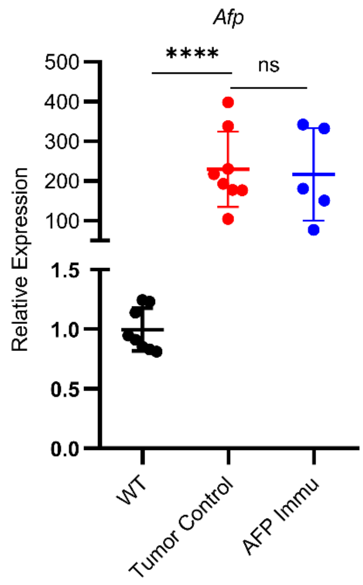

Supplementary Figure 4. AFP expression in the immunized c-MYC/Mcl1 tumors is still intact. WT is used as a negative control. ANOVA analysis was used. Abbreviation: WT, Immu, immunization Ns, no significance, ****P < 0.0001.

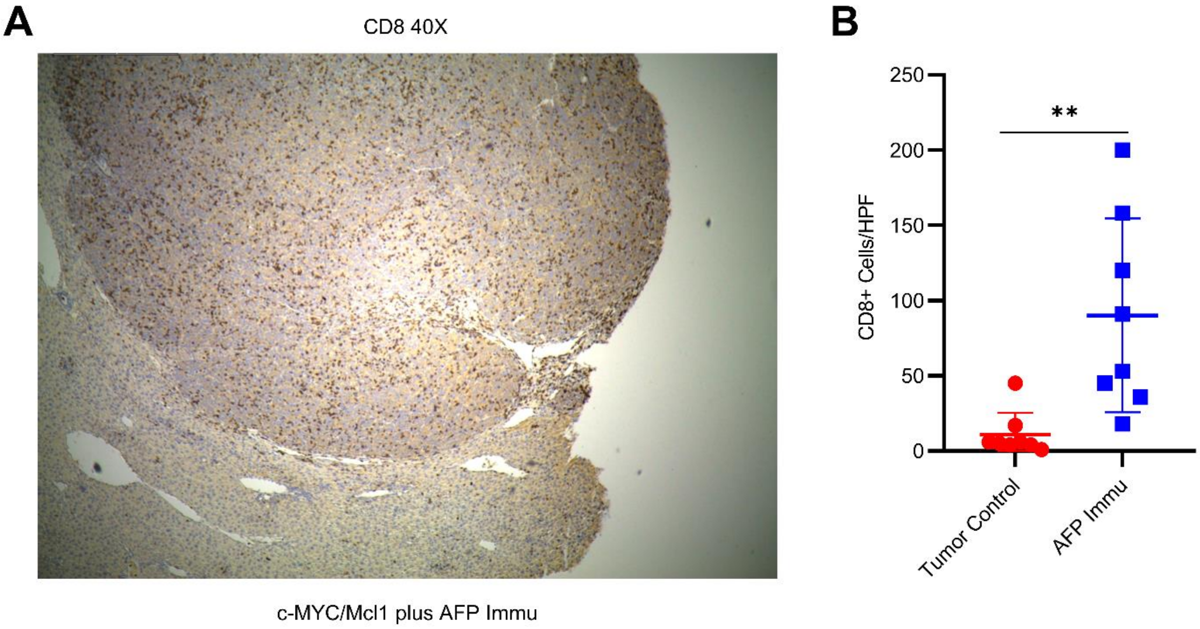

Supplementary Figure 5. AFP immunization dramatically increases CD8+T cell infiltration in early-stage tumors. Representative CD8 staining of c-MYC/Mcl1 with AFP immunization in early-stage (A) and its quantification results compared to the control group (B, n=8 fields). Original magnification: 40X. Unpaired two-tailed student's t-test was used in (B). ** $p \le 0.01$. Original magnification: 40X. Abbreviations: HPF, high power field; Immu, immunization.

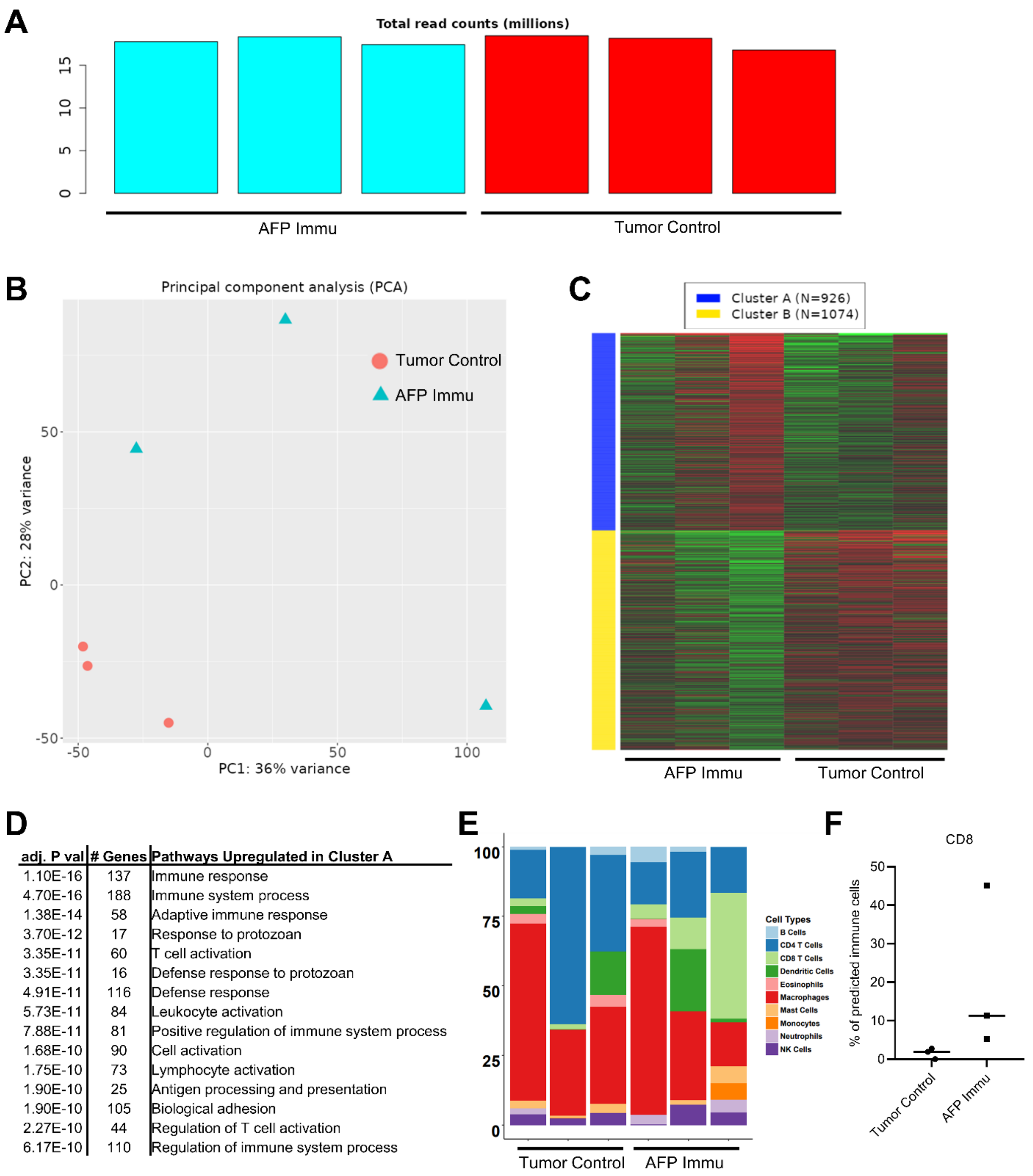

Supplementary Figure 6: RNAseq of c-MYC/Mcl1 tumors with or without AFP immunization. Bulk mRNA from tumors (n=3) with or without AFP immunization was subjected to whole exome RNAseq. Data were analyzed on the iDEP.96 and ImmuCC platforms. (A) Total read counts for each sample (in millions). (B) PCA plot between tumor control and AFP immunization groups. (C)

K-means supervised clustering between the investigated groups. (D) Significantly upregulated GO pathways based on clustering in (C). (E) Predictions of percentages of major immune cell populations from *in silico* cell deconvolution using the ImmuCC platform between the investigated groups. (F) Quantification of CD8+ T cells from (E). Abbreviations: Immu, immunization.

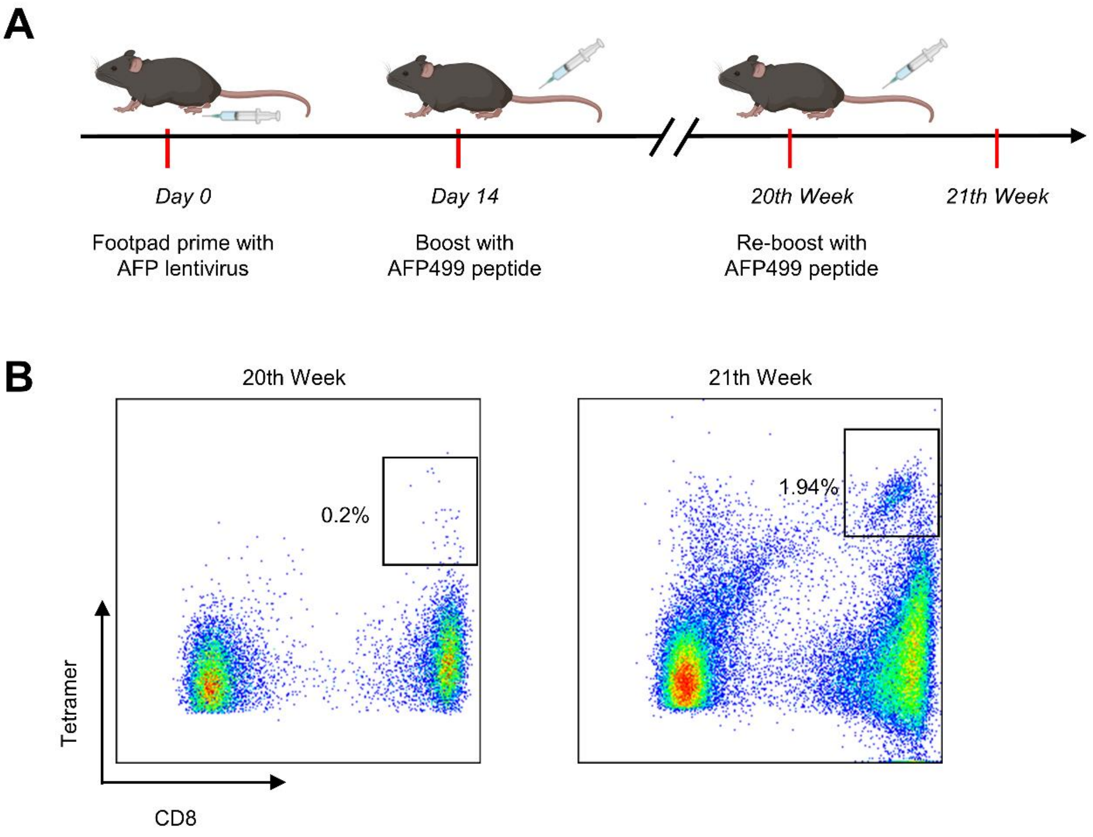

Supplementary Figure 7. AFP antigen-specific CD8+ T cells possess cell memory. (A) Study design. (B) AFP antigen-specific CD8+ T cells (AFP499+) were re-boosted (right panel) by AFP499 peptide at 20 weeks post-induction (left panel).

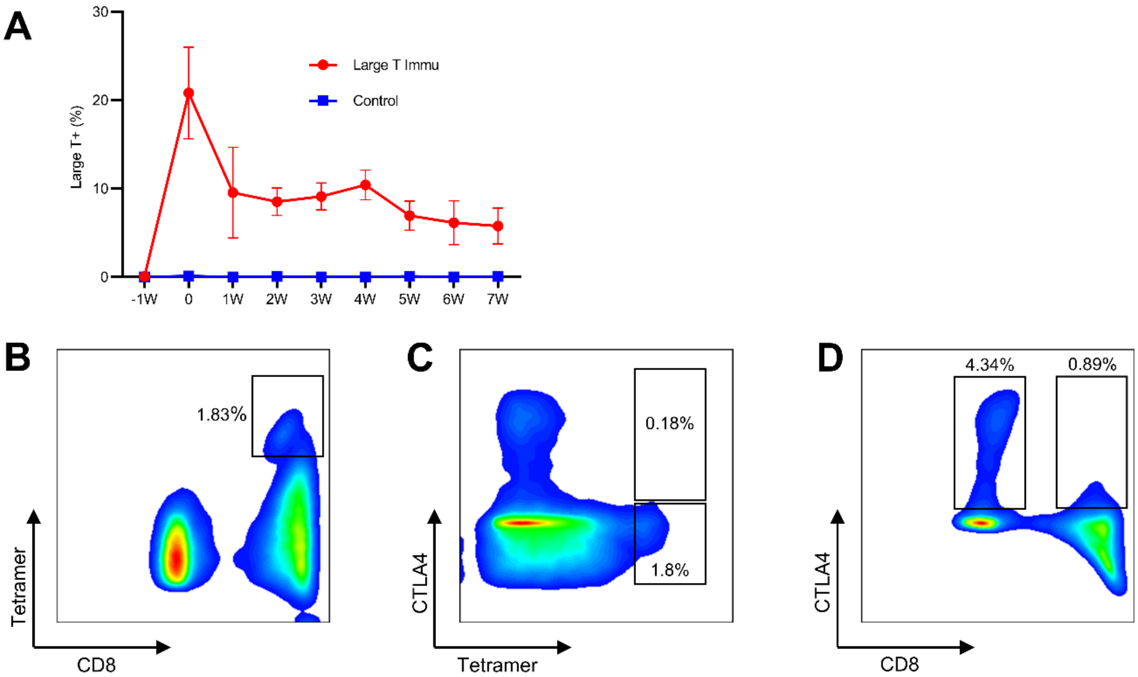

Supplementary Figure 8. AFP antigen-specific T cells do not express CTLA4. (A) Decrease of Large T peptide-specific CD8+ T cells (Large T+) in the Large T immunized mice. (B-D) Representative flow cytometry results of an AFP immunized mouse. (B-D) are gated from CD3+ T cells. The AFP antigen-specific CD8+ T cells (B) have no CTLA4 expression (C), whereas the CTLA4 is mainly expressed in the CD8- T cells, namely, CD4+ T cells. Abbrevations: CTLA4, cytotoxic T-lymphocyte-associated protein 4; Immu, immunization.

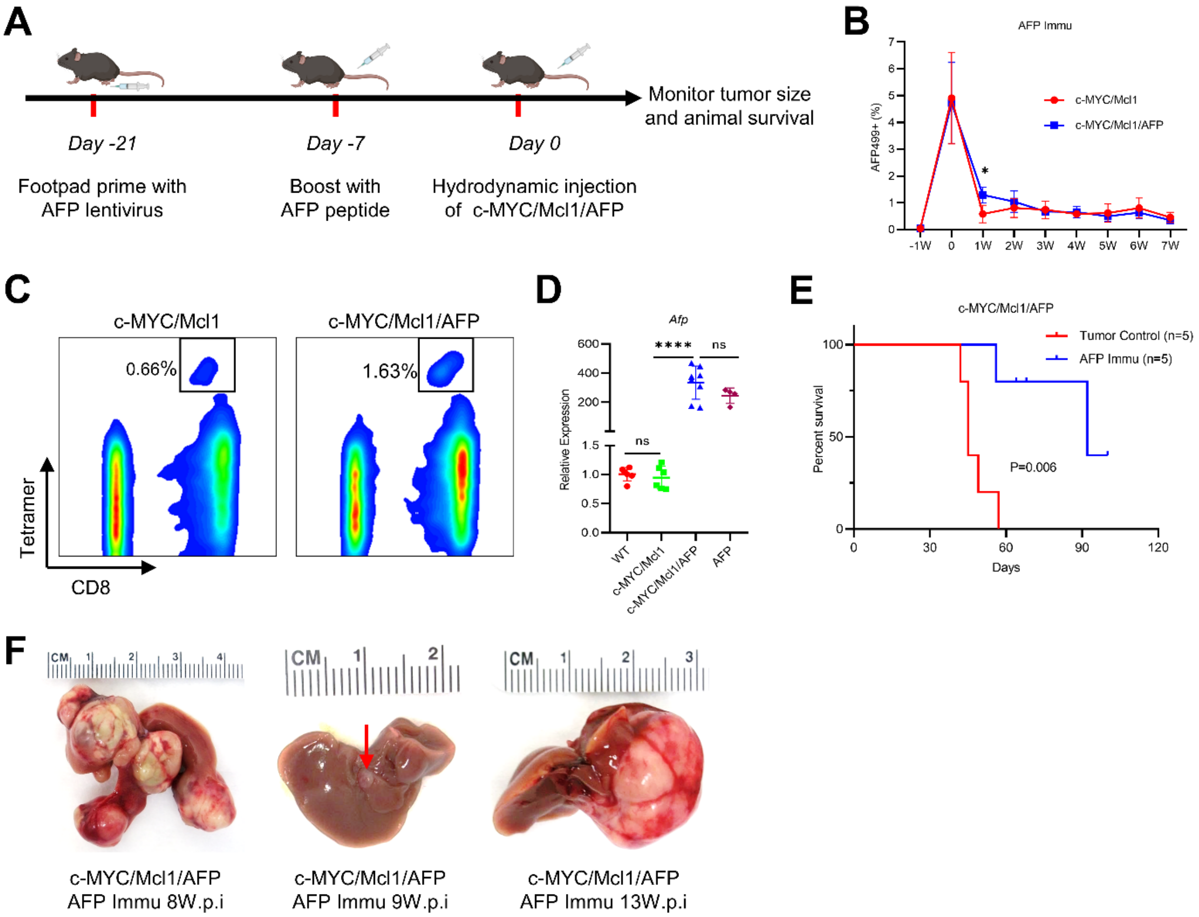

Supplementary Figure 9. Co-expression of AFP and c-MYC/Mcl1 hydrodynamical injection still results in tumor growth. (A) Study design. (B) Dynamic changes of AFP499+ CTLs in c-MYC/Mcl1 or c-MYC/Mcl1/AFP immunized mice. (C) Representative flow cytometry results from (B) at the first-week post-AFP499+ CTL induction. (D) Relative expression of Afp in the liver of c-MYC/Mcl1, c-MYC/Mcl1/AFP, and AFP only after 1 week of hydrodynamic injection. WT is used as a negative control. (E) Survival curve of the c-MYC/Mcl1/AFP with or without AFP immunization. (F) Pictures of representative livers from (E); the red arrow indicates a small tumor nodule. Unpaired two-tailed student's t-test was used in (B), ANOVA analysis was used in (D), kaplan-Meier test was used for survival analysis in (E). Ns, no significance, *P < 0.05, ***P < 0.001. Abbreviation: W.p.i, weeks post-injection; Immu, immunization.

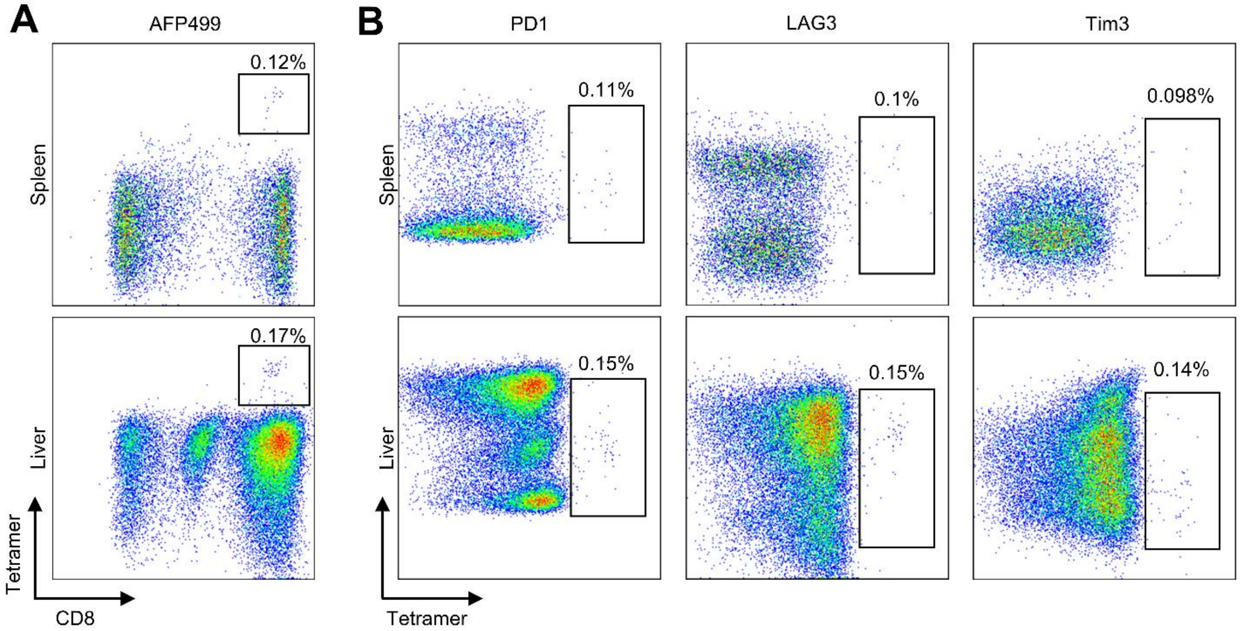

Supplementary Figure 10. AFP antigen-specific T cells still co-express exhaustion markers in moribund mice. (A). AFP antigen-specific CD8+ T cells (AFP499) can still be detected in end-stage liver tumors and spleen. (B) Co-expression of exhaustion markers, PD1, LAG3, and Tim3 on AFP499 cells. Abbreviations: AFP499, AFP499 antigen-specific CD8+ T cells; PD1, programmed cell death protein 1; LAG3, lymphocyte-activation gene 3; Tim3, T cell immunoglobulin and mucin protein 3.

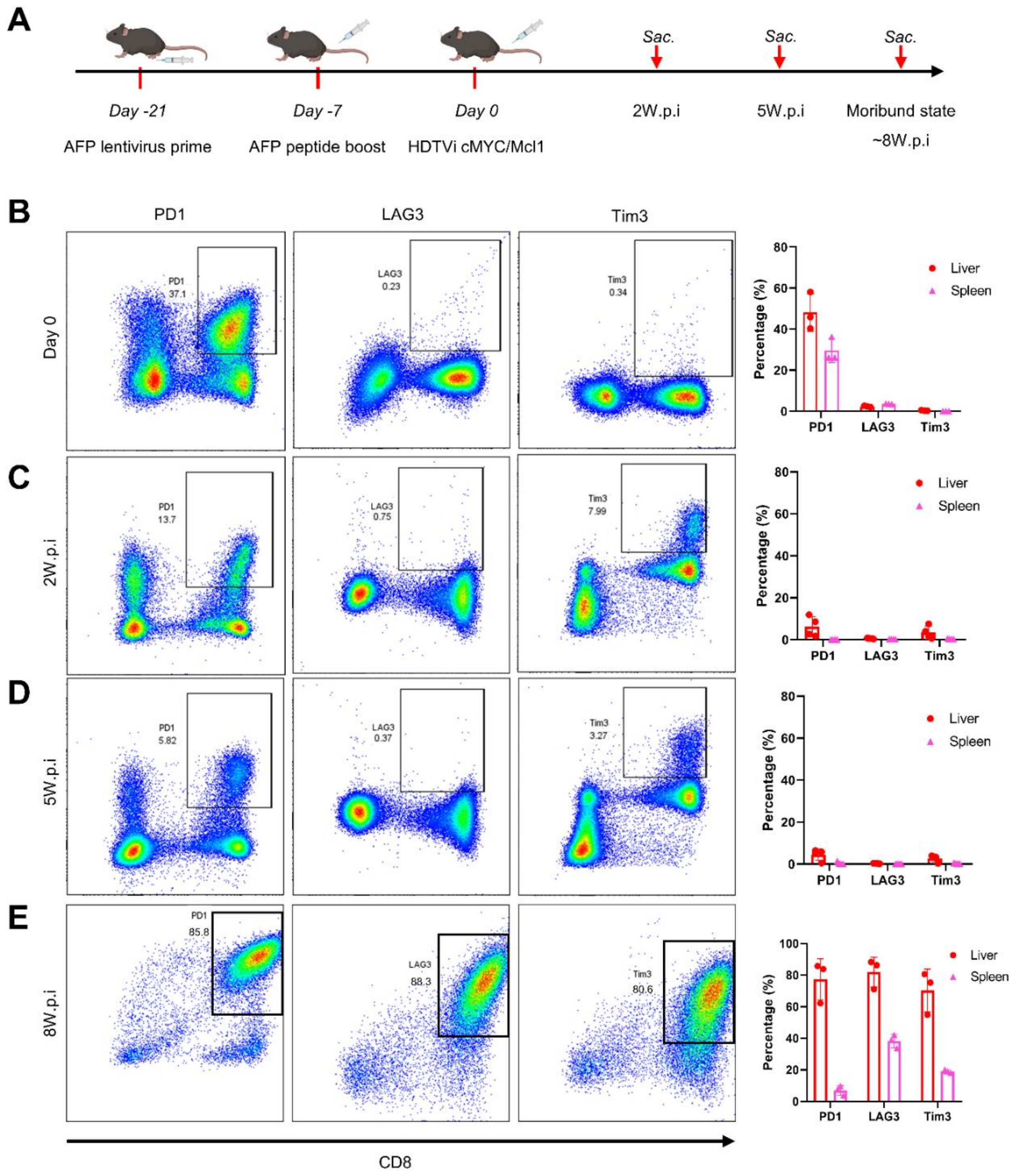

Supplementary Figure 11: Time course assessment of exhaustion markers on CD8+ T cells in immunized mice bearing c-MYC/Mcl1 tumors. (A) Study design. (B)-(E) Gating and quantification of percentage of CD3+CD8+ T cells expressing PD1, LAG3, or Tim3 at indicated various time points and organs. Unpaired two-tailed student's t-test was used in (B-E). Abbreviations: HDTVi, hydrodynamic tail vein injection; W.p.i, weeks post-injection; PD1, programmed cell death protein 1; LAG3, lymphocyte-activation gene 3; Tim3, T cell immunoglobulin and mucin protein 3.

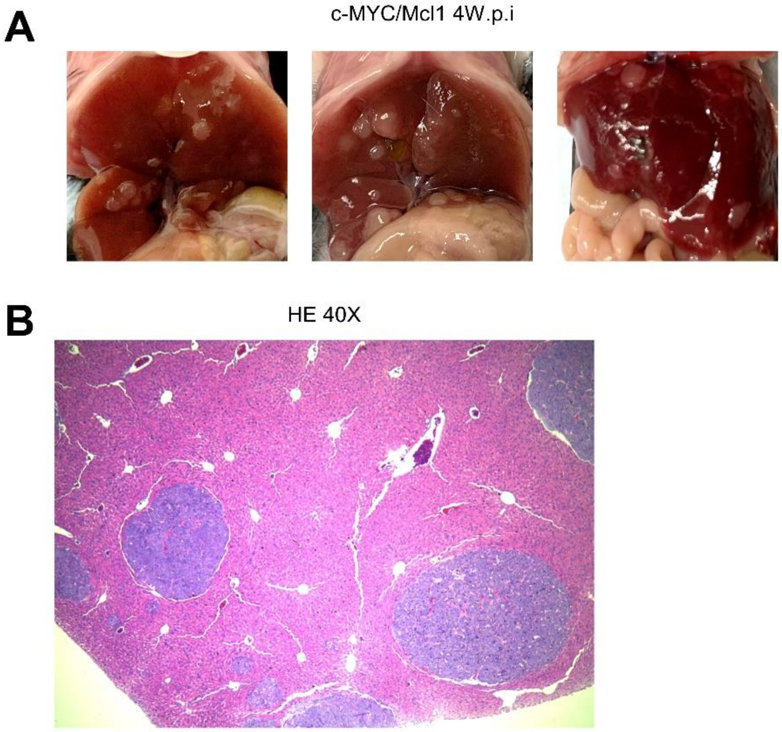

Supplementary Figure 12. Tumor nodules form 4 weeks after c-MYC/Mcl1 hydrodynamic injection. (A) Gross images of tumor nodules 4 weeks after c-MYC/Mcl1 hydrodynamic injection. (B) HE staining of tumor nodules from (A). Original magnification: 40X. Abbreviations: W.p.i, weeks post-injection; HE, hematoxylin and eosin staining.

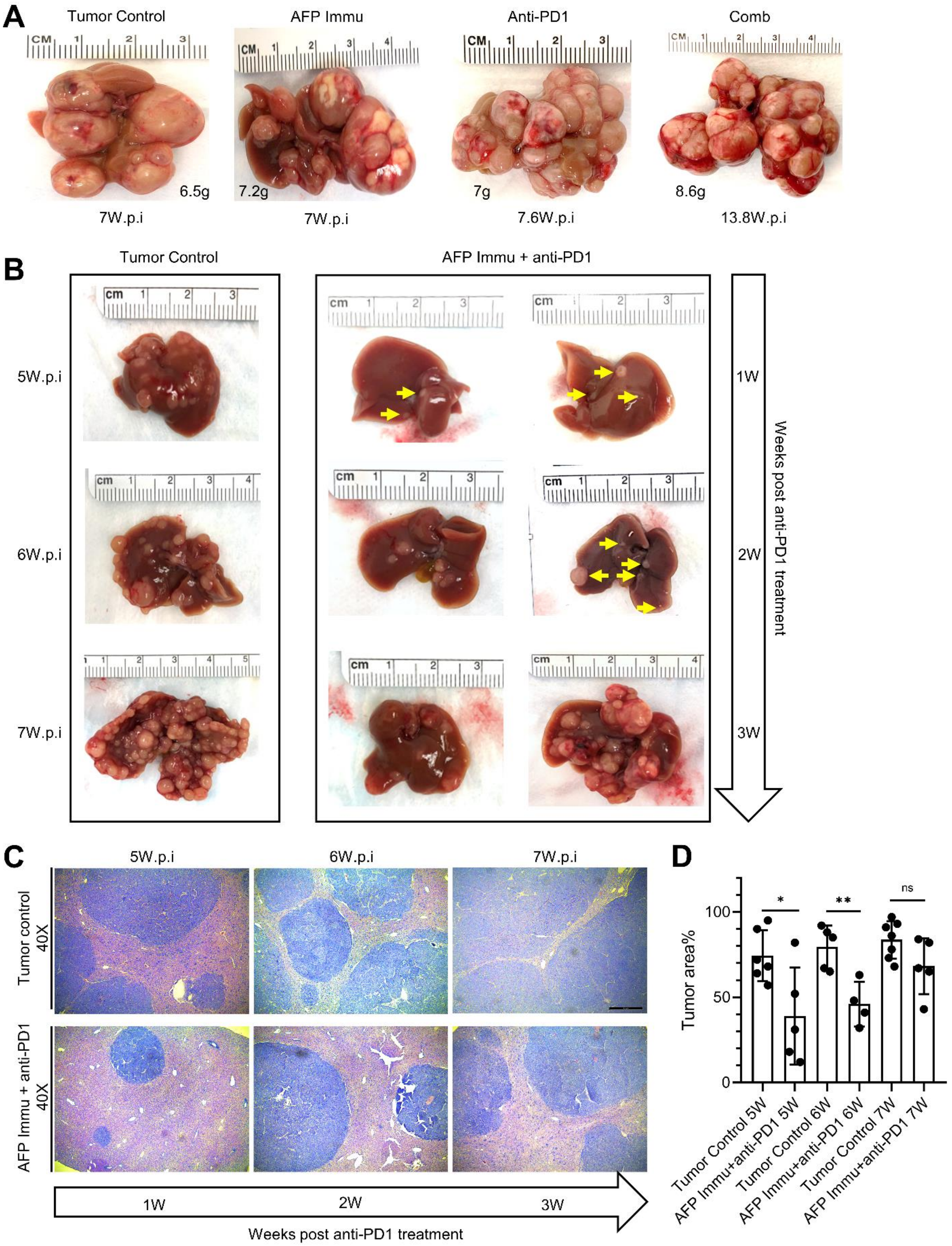
A
Tumor Control
AFP Immu
Anti-PD1
Comb
6.5g
7.2g
7g
8.6g
7W.p.i
7W.p.i
7.6W.p.i
13.8W.p.i
B
Tumor Control
AFP Immu + anti-PD1
5W.p.i
6W.p.i
7W.p.i
1W
2W
3W
Weeks post anti-PD1 treatment
C
5W.p.i
6W.p.i
7W.p.i
Tumor control 40X
AFP Immu + anti-PD1 40X
1W
2W
3W
Weeks post anti-PD1 treatment
D
Tumor area%
100
50
0
*
**
ns
Tumor Control 5W
AFP Immu+anti-PD1 5W
Tumor Control 6W
AFP Immu+anti-PD1 6W
Tumor Control 7W
AFP Immu+anti-PD1 7W

Supplementary Figure 13. Representative gross image of each group of treated tumors. (A) Pictures of representative livers from (Figure 5B); numbers indicate the liver weight and weeks from hydrodynamic injection to sacrifice date for that particular mouse. (B) The time course study panel of 5, 6, or 7 weeks for the tumor control and AFP immunization combined with anti-PD1 to treat c-MYC/Mcl1-induced HCC tumors. The yellow arrowheads indicate the small tumor nodules in the livers. (C) Representative HE-stained images of livers in (B). Original magnification: 40X. (D) Quantification of the tumor area from (C), unpaired two-tailed student's t-test was used. Ns, no significance, *P < 0.05, **P < 0.01. Abbreviation: W.p.i, weeks post-injection; W, week(s), Comb, combination therapy; Immu, immunization.

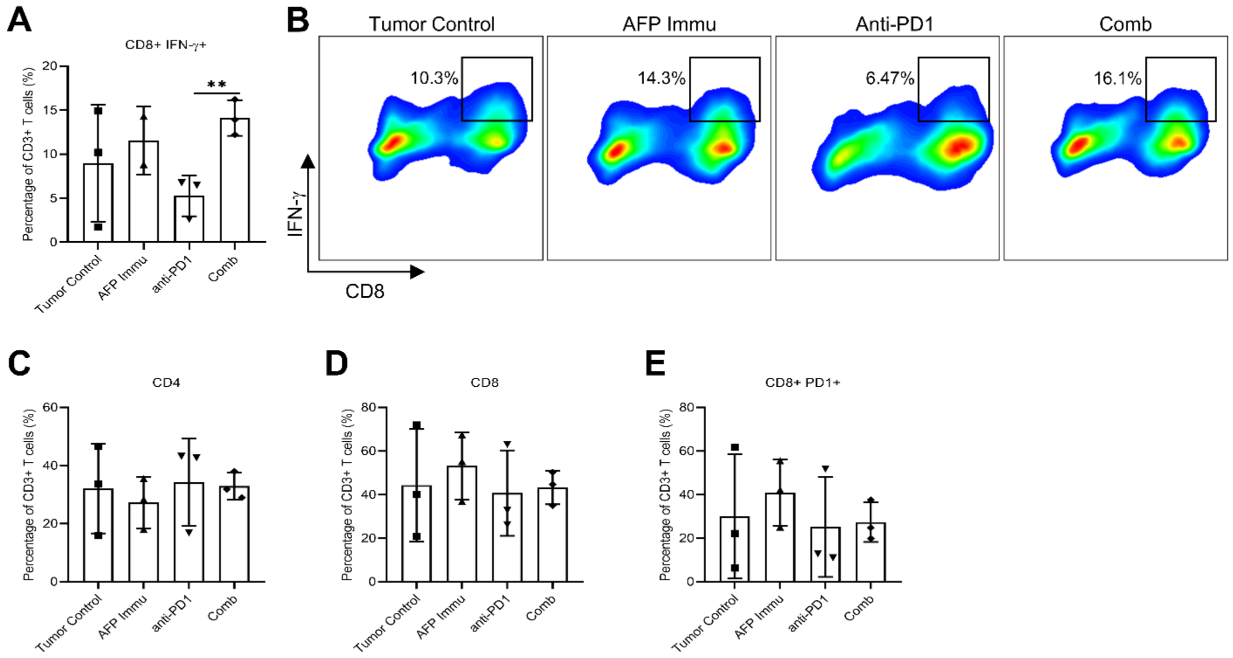

Supplementary Figure 14. T cell subpopulations in liver tumors of the anti-PD1 combination therapy. (A-B) Quantification of the CD8+IFN-γ+ T cells (A) and their corresponding representative flow cytometry results (B) in each group. (C-E) Percentages of CD4+, CD8+, CD8+PD1+ T cell subpopulations in the various groups tested. ANOVA analysis was used, ** $p \leq 0.01$. Abbreviations: Comb, combination therapy; Immu, immunization.

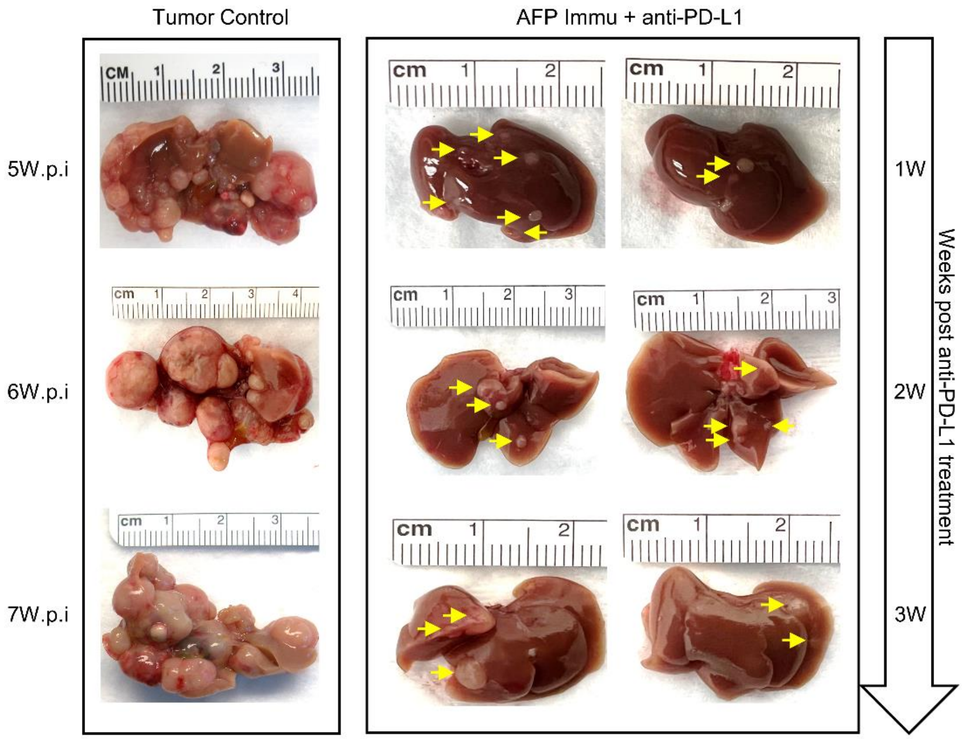

Supplementary Figure 15. Time course study panel of 5, 6, and 7 weeks for the tumor control and AFP immunization combined with anti-PD-L1 to treat c-MYC/Mcl1 induced HCC tumors. The yellow arrowheads are used to increase the recognition of the small tumor nodules in the livers. Abbreviations: W.p.i, weeks post-injection, W, week(s); Immu, immunization.

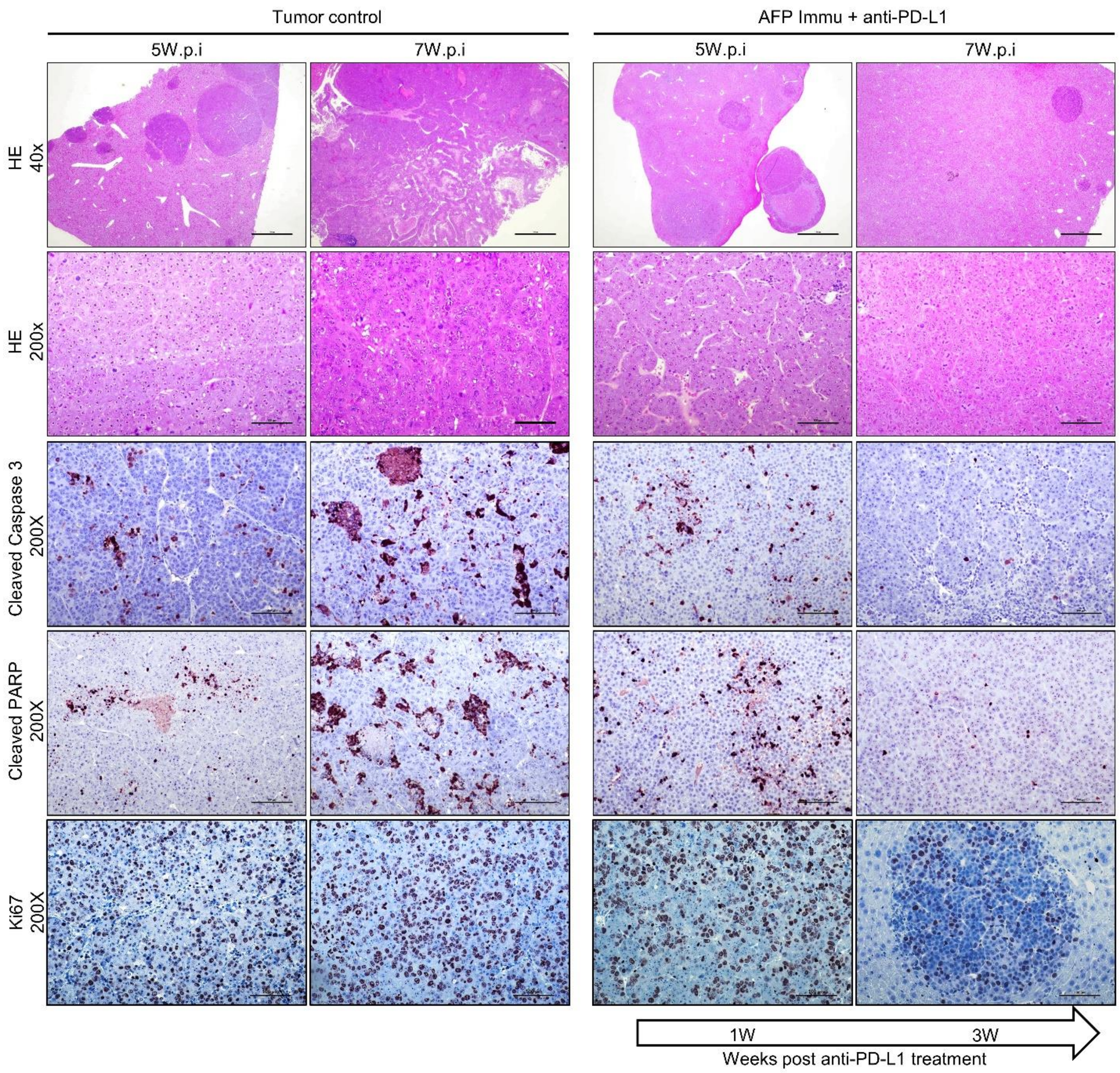

Supplementary Figure 16. Histology, apoptosis, and proliferation features of c-MYC/Mcl1 control and AFP immunization/anti-PD-L1 treated tumors. Hematoxylin and eosin staining (HE) shows that tumors in the two cohorts of mice are morphologically identical. Apoptosis was assessed by cleaved Caspase 3 and Cleaved PARP staining, whereas proliferation was determined via Ki67 immunohistochemistry. Note the low apoptosis rate in the lesions after 3 weeks of treatment (7 weeks post-HDTVi injection). Original magnification: 40X and 200X. Abbreviation: W.p.i, weeks post-injection.

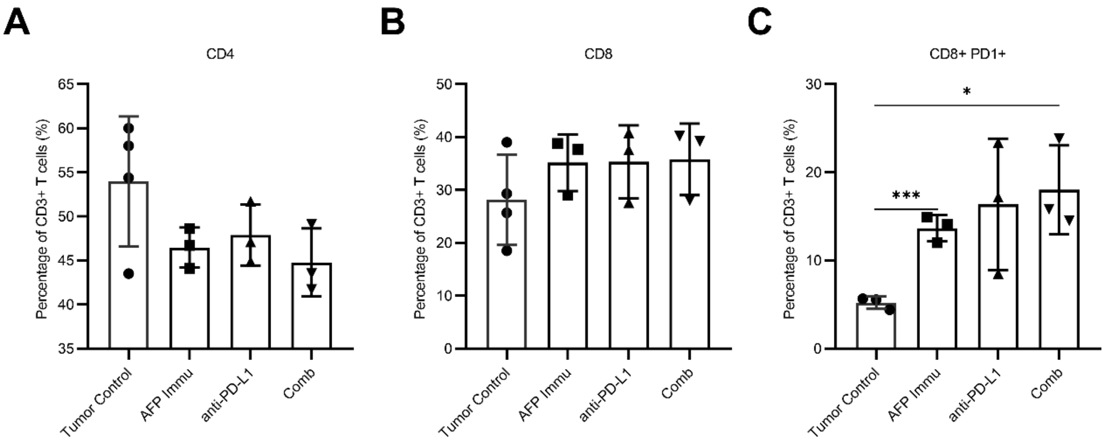

Supplementary Figure 17. T cell subpopulations in liver tumors of the anti-PD-L1 combination therapy. (A-C) Percentages of CD4+, CD8+, and CD8+PD1+ T cell subpopulations in the various groups tested. ANOVA analysis was used, *P < 0.05, ***P < 0.001. Abbreviation: Comb, combination therapy; Immu, immunization.

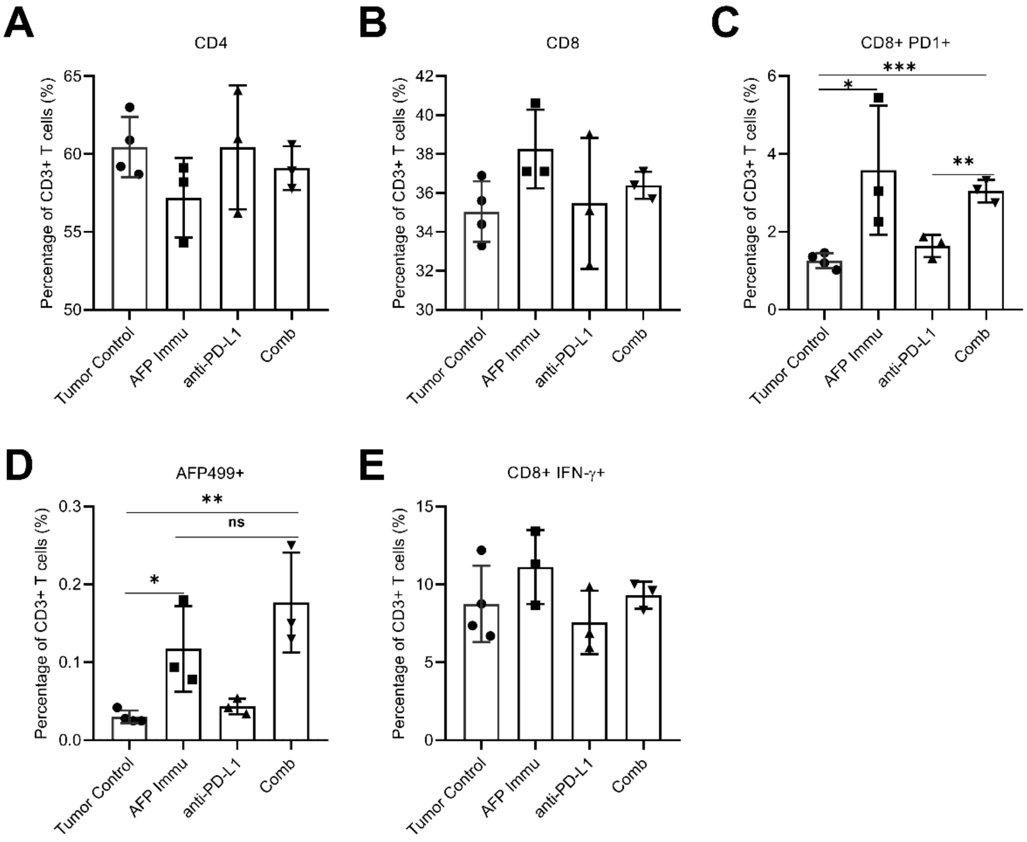

Supplementary Figure 18. T cell subpopulations in the spleen of the anti-PD-L1 combination therapy. (A-E) Percentage of CD4+, CD8+, CD8+PD1+, AFP499+ CTLs, and CD8+IFN-γ+ T cell subpopulations in the spleens from (Figure 6B). ANOVA analysis was used. Ns, no significance, *P < 0.05, **P < 0.01, ***P < 0.001. Abbreviations: Comb, combination therapy; Immu, immunization.

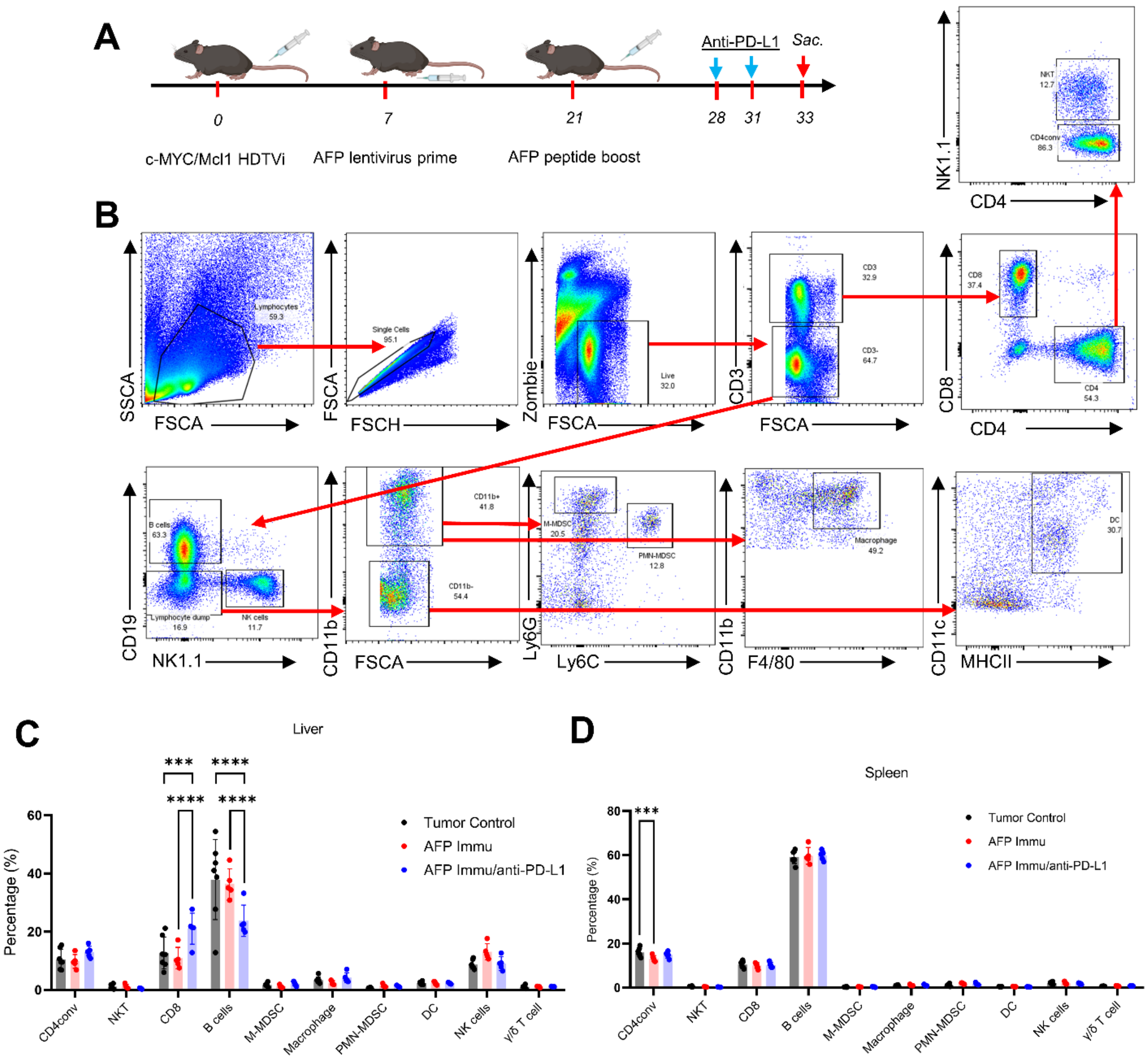

Supplementary Figure 19: Flow cytometry immune profiling of c-MYC/Mcl1 liver tumors subjected to combination immunotherapy. (A) Study design. (B) Gating strategy and quantification of the frequency of major immune cell types in the (C) liver and (D) spleen from mice with c-MYC/Mcl1 tumors. ANOVA analysis was used in (C) and (D). Data are shown as mean ± s.d. Ns p > 0.05, * p ≤ 0.05, ** p ≤ 0.01, *** p ≤ 0.001, **** p ≤ 0.0001. Abbreviations: HDTVi, hydrodynamic tail vein injection; Immu, immunization; MDSC, myeloid-derived suppressor cell; M-MDSC, monocytic-MDSC; PMN-MDSC, polymorphonuclear-MDSC; NKT, natural killer T cell; NK, natural killer cell. CD4 conv, CD4+ conventional T cell.

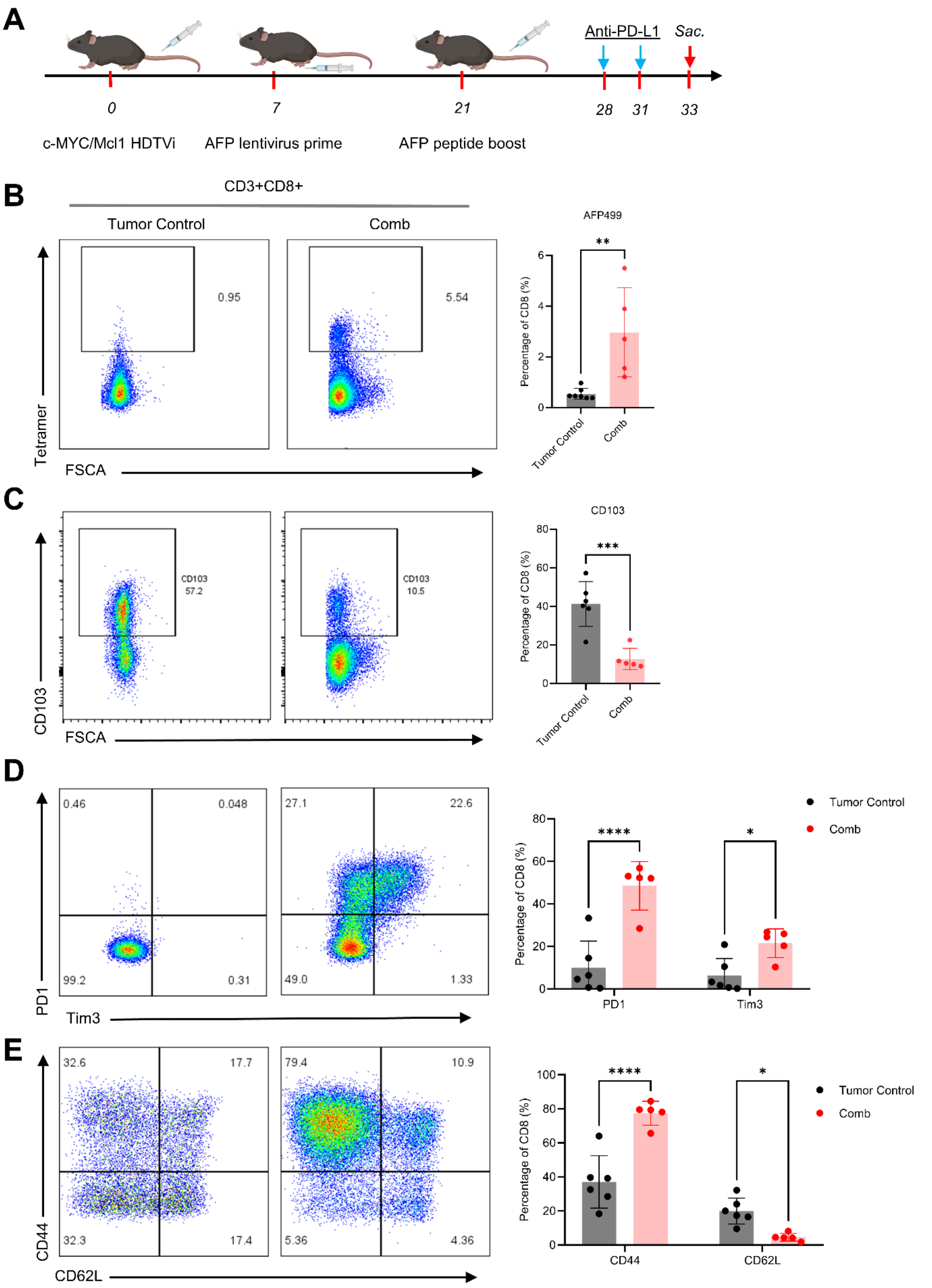
A
Anti-PD-L1
Sac.
0
7
21
28
31
33
c-MYC/Mcl1 HDTVi
AFP lentivirus prime
AFP peptide boost
B
CD3+CD8+
Tumor Control
Comb
0.95
5.54
Tetramer
FSCA
AFP499
Percentage of CD8 (%)
**
C
CD103
57.2
CD103
10.5
CD103
FSCA
CD103
***
D
0.46
0.048
99.2
0.31
27.1
22.6
49.0
1.33
PD1
Tim3
Tumor Control
Comb
****
*
E
32.6
17.7
32.3
17.4
79.4
10.9
5.36
4.36
CD44
CD62L

Supplementary Figure 20: CD8 subsets from c-MYC/Mcl1 tumor-bearing livers of mice treated with the combination of AFP immunization and anti-PD-L1. (A) Study design. (B)-(E) Percentage of CD8+ T cells expressing TCR recognizing AFP499-specific T cells (B), the liver residency marker CD103 (C), exhaustion markers PD1 and Tim3 (D), and activation (CD44+CD62L-) and naïve (CD62L+CD44-) markers (E). Unpaired two-tailed Student's *t*-test was used in (B) and (C). ANOVA analysis was used in (D) and (E). ns $p > 0.05$, * $p \leq 0.05$, ** $p \leq 0.01$, *** $p \leq 0.001$, **** $p \leq 0.0001$. Abbreviations: AFP499, AFP499 antigen-specific CD8+ T cells; HDTVi, hydrodynamic tail vein injection; Immu, immunization; Sac., sacrifice; PD1, programmed cell death protein 1; Tim3, T cell immunoglobulin and mucin protein 3; Comb, combination therapy

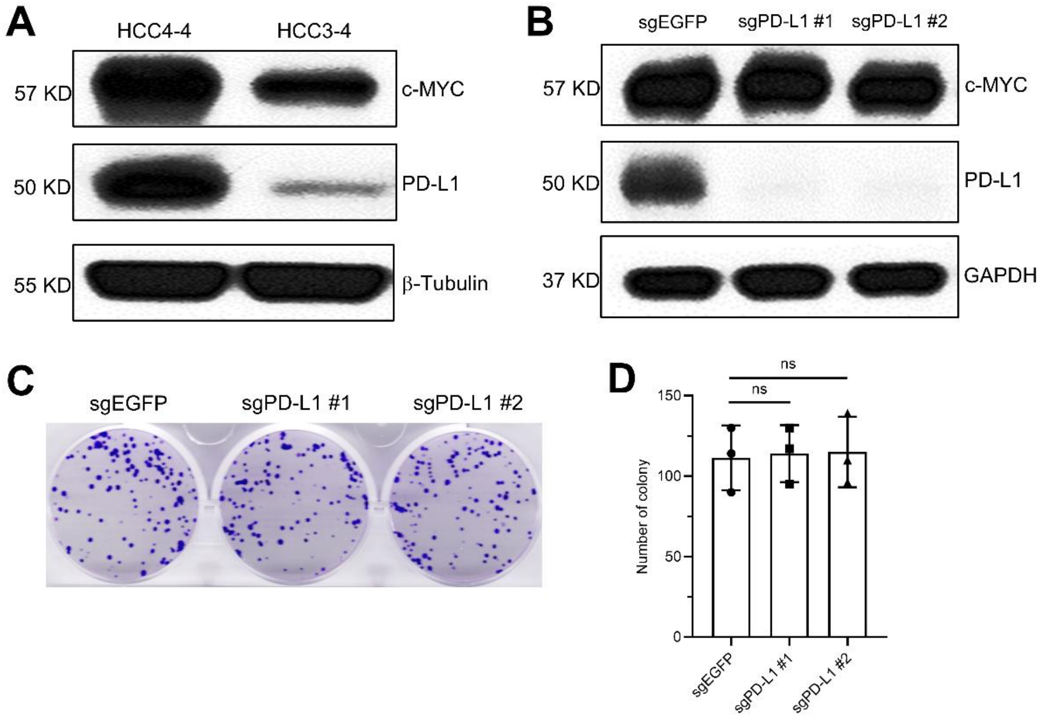

Supplementary Figure 21. Depletion of PD-L1 in the HCC tumor cells does not affect their growth. (A) Western blot analysis of c-MYC and PD-L1 expression in two c-MYC-overexpressing HCC cells, HCC4-4 and HCC3-4. (B-D) Deletion of PD-L1 in the c-MYC-overexpressing HCC4-4 cells, which display high levels of PD-L1 (B), does not affect their cell growth rate (C-D). sgEGFP was used as a non-target control. ANOVA analysis was used in (D). Ns, no significance.

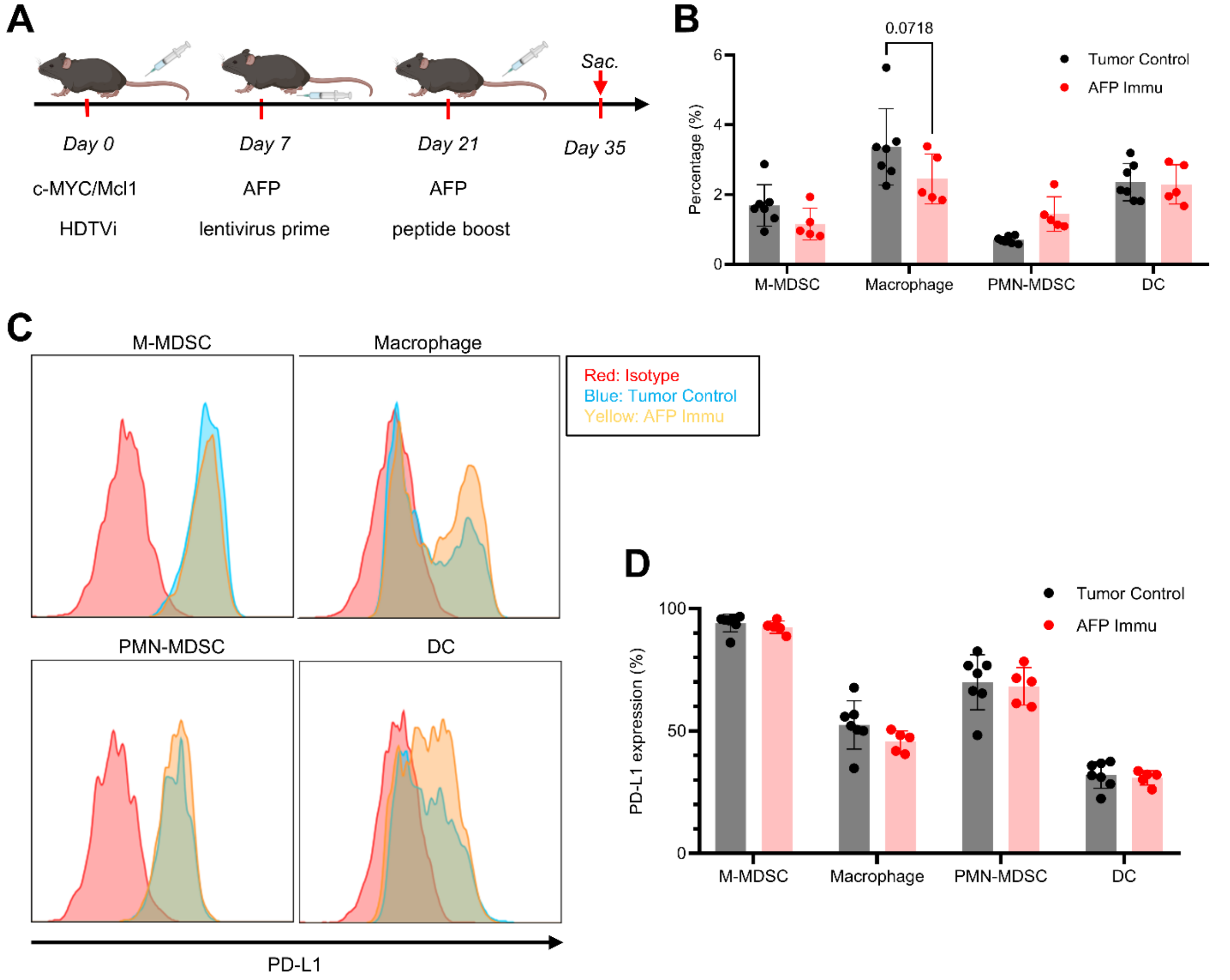

Supplementary Figure 22: PD-L1 expression on myeloid cells from c-MYC/Mcl1 tumor-bearing mice following AFP immunization. (A)-(B) Study design and myeloid cell frequency for the c-MYC/Mcl1 tumors with or without AFP immunization. (C)-(D) Flow cytometry histograms (C) and quantification of PD-L1 (D) on indicated myeloid cells in (B). PD-L1 was gated off of isotype control. Unpaired two-tailed student's t-test was used in (B) and (D) between respective groups. Data are shown as mean ± s.d. Abbreviations: HDTVi, hydrodynamic tail vein injection; Immu, immunization; Sac., sacrifice; PD-L1, programmed death ligand 1; DC, dendritic cell; MDSC, myeloid-derived suppressor cell; M-MDSC, monocytic myeloid-derived suppressor cell; PMN-MDSC, polymorphonuclear-MDSC.

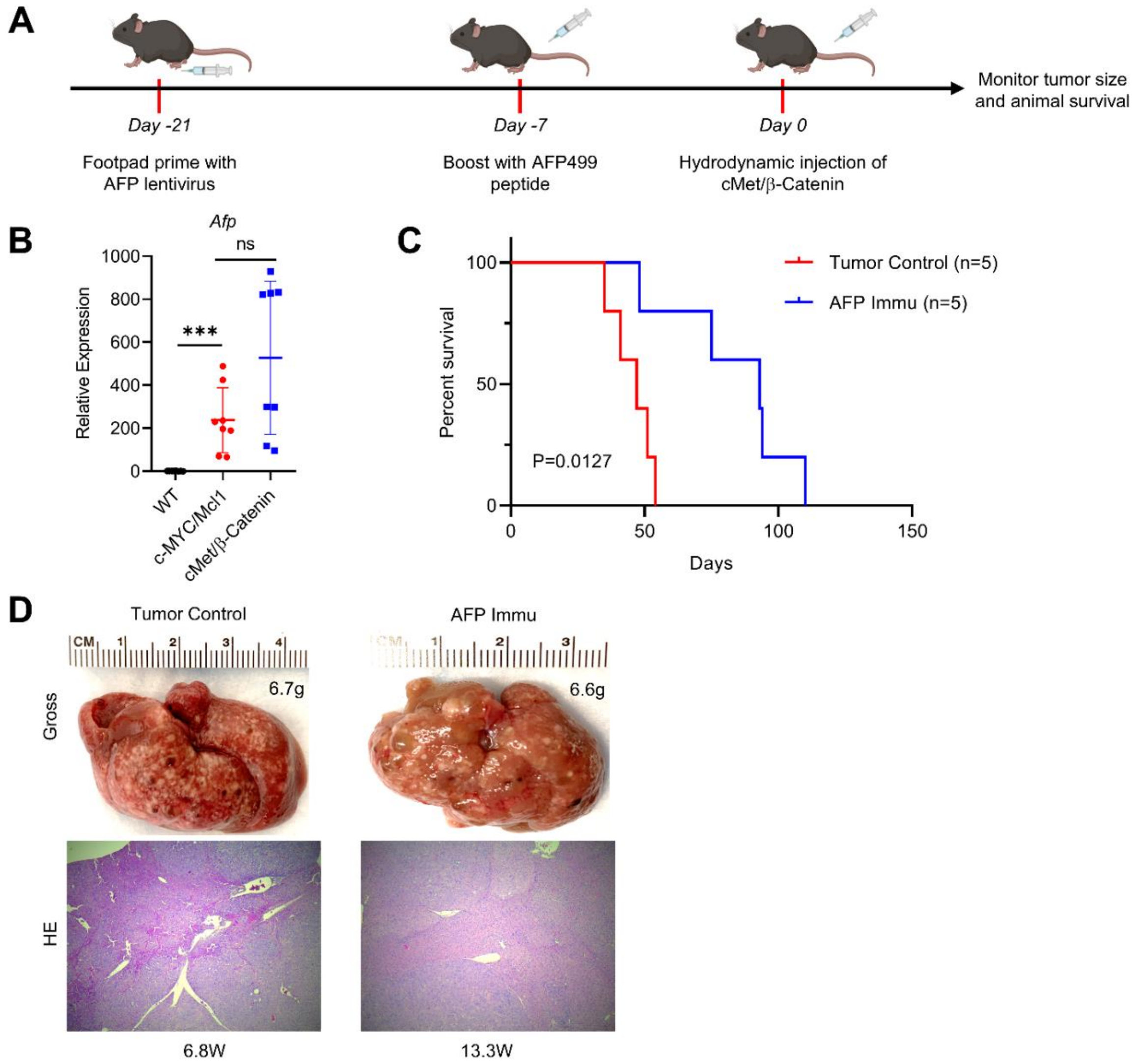

Supplementary Figure 23. AFP immunization prevents the autologous HCC formation induced by cMet/β-Catenin hydrodynamic injection. (A) Study design of AFP immunization and cMet/β-Catenin hydrodynamic injection. (B) AFP mRNA expression in c-MYC/Mcl1 and cMet/β-Catenin HCC tumors, WT is used as a negative control. Ns, no significance, ***P < 0.001. (C) Survival curve of the Control and AFP immunized group in the cMet/β-Catenin mouse model. (D) Representative gross images and HE results of the investigated groups. Original magnification: 40X. ANOVA analysis was used in (B), Kaplan-Meier test was used for survival analysis in (C). The numbers indicate the liver weight and weeks from plasmids injection to the sacrifice date for that particular mouse. Abbreviation: Immu, immunization.

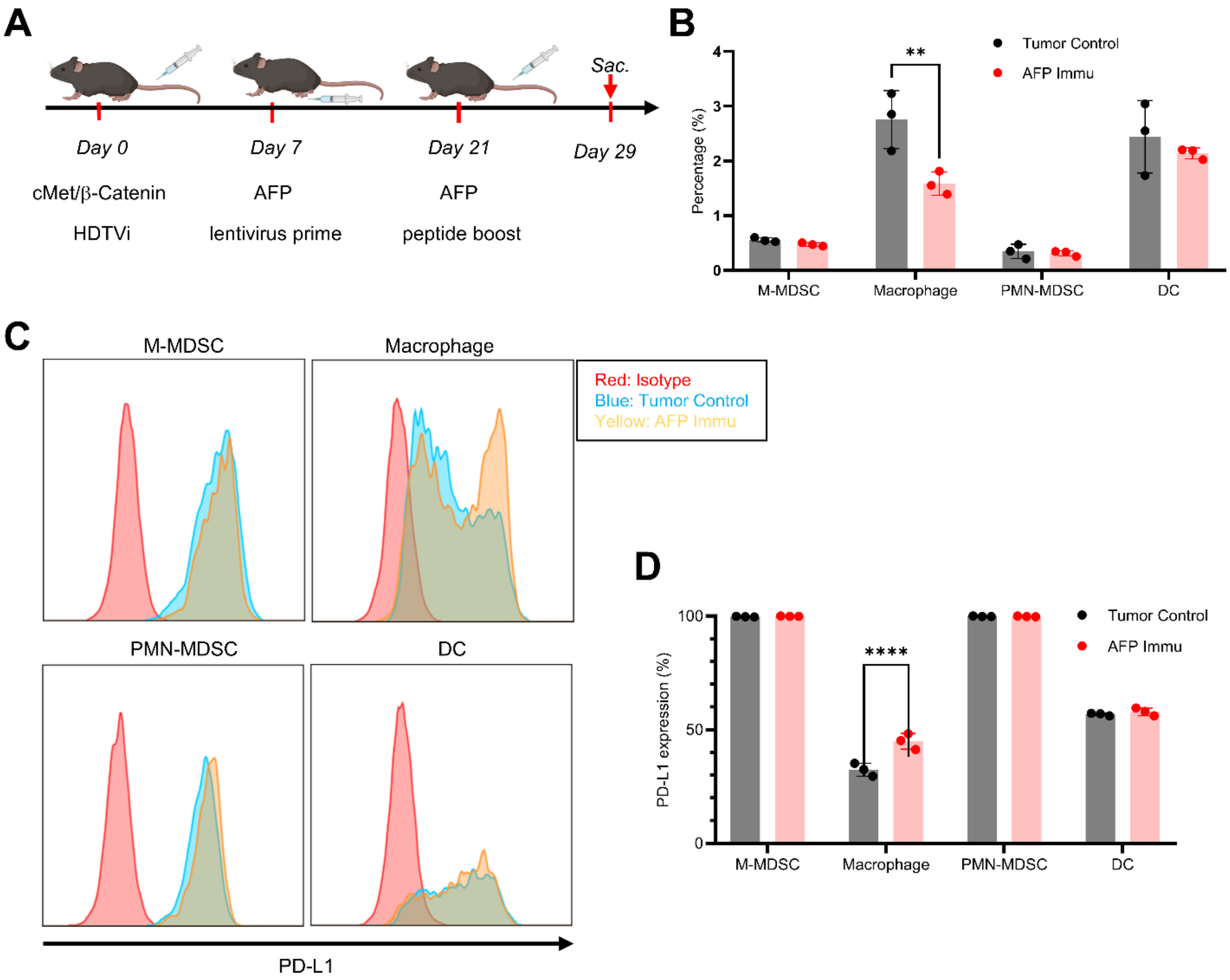

Supplementary Figure 24: PD-L1 expression on myeloid cells from cMet/β-catenin tumor-bearing mice following AFP immunization. (A)-(B) Study design and myeloid cell frequency for the cMet/β-catenin tumors with or without AFP immunization. (C)-(D) Flow cytometry histograms (C) and quantification of PD-L1 (D) on indicated myeloid cells in (B). PD-L1 was gated off of isotype control. Unpaired two-tailed student's t-test was used in (B) and (D) between respective groups. Data are shown as mean ± s.d. **** $p \leq 0.0001$. Abbreviations: HDTVi, hydrodynamic tail vein injection; Immu, immunization; Sac., sacrifice; PD-L1, programmed death ligand 1; DC, dendritic cell; MDSC, myeloid-derived suppressor cell; M-MDSC, monocytic myeloid-derived suppressor cell; PMN-MDSC, polymorphonuclear-MDSC.

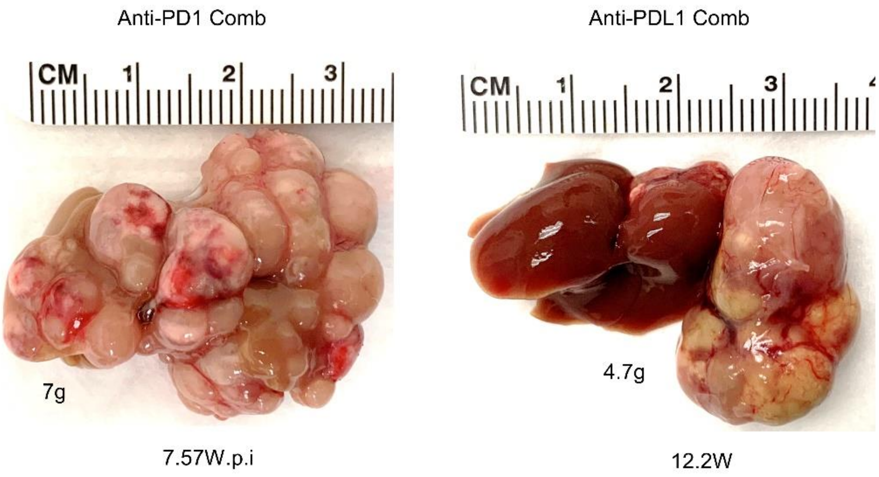

Supplementary Figure 25: Liver tumors from AFP-immunized mice treated with anti-PD-1 vs. anti-PDL1. Represented pictures were extracted from Figure 6 and Supplementary Figure 9. The numbers indicate the liver weight and weeks from plasmids injection to the sacrifice date for that particular mouse.

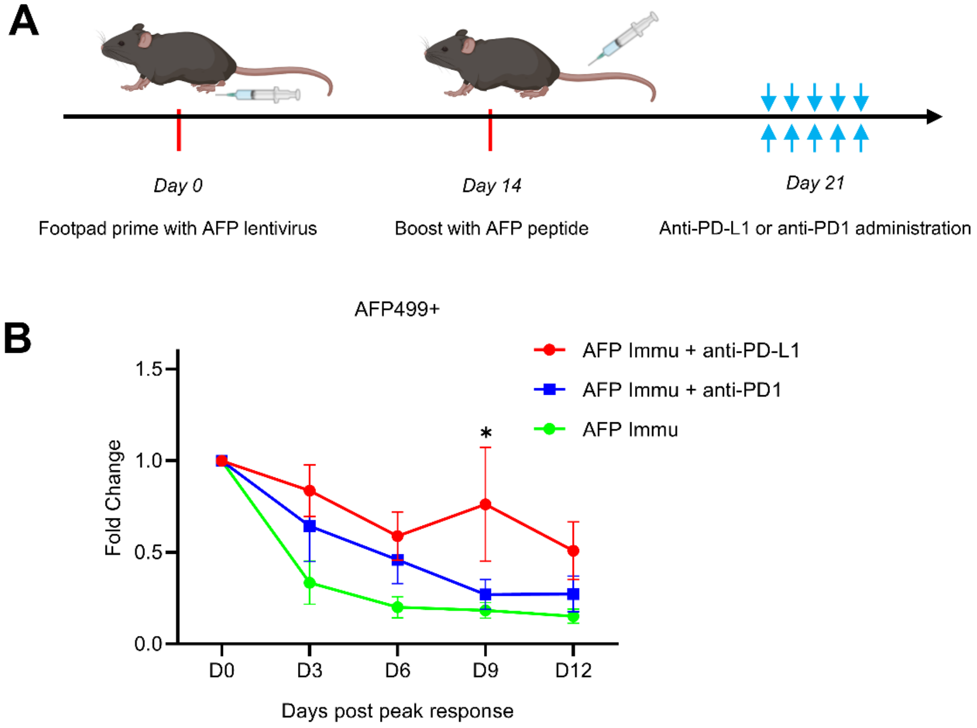

Supplementary Figure 26. Anti-PD1 and anti-PD-L1 administration prolongs the survival of AFP499+ CTLs. (A) Study design. (B) Dynamic change of AFP499+ CTLs after anti-PD1 and anti-PD-L1 treatment. Data shows a significant difference in the AFP499+ CTLs on Day 9 in the AFP immunization plus anti-PD-L1 group compared to the AFP immunized-only group. The y-axis fold change was obtained by the percentage of AFP499+ CTLs on D0, D3, D6, D9, and D12 compared to the percentage of AFP499+ CTLs on D0. ANOVA analysis was used in (B), *P < 0.05. Abbreviations: AFP499+, AFP499 peptide-specific CD8+ T cells; Immu, immunization.

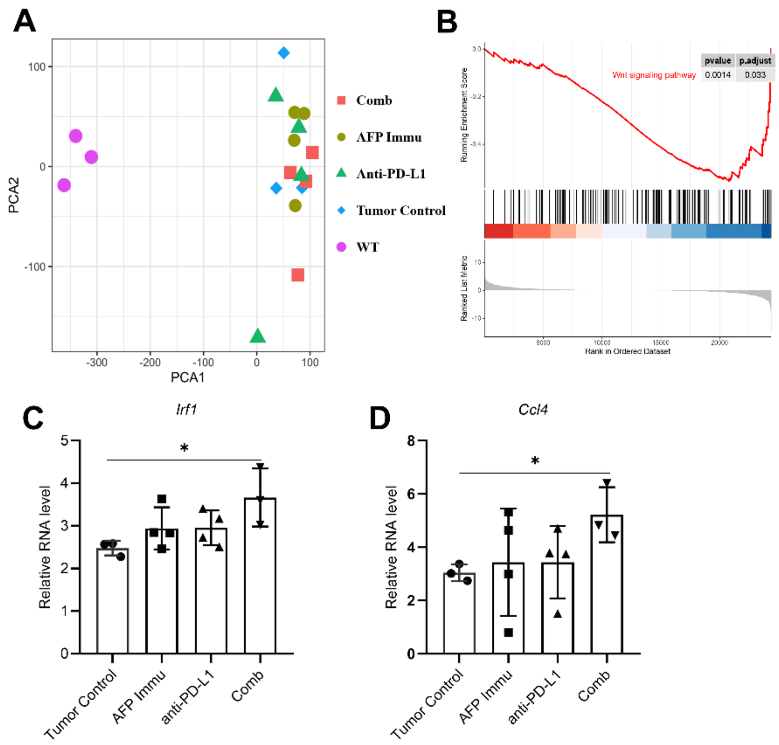

Supplementary Figure 27. Genes related to CD8+ T cells identification through RNA-sequencing. (A) PCA plot of all the groups tested. (B). GSEA of the *Ctnnb1* gene signature in the Comb group and tumor control group. (C-D). Relative expression of *Irf1* (C) and *Ccl4* (D) in liver tumors from anti-PD-L1 combination therapy. ANOVA analysis was used in (C) and (D), $^{*}P < 0.05$. Abbreviation: Comb, combination therapy; Immu, immunization; NES, normalized enrichment score.

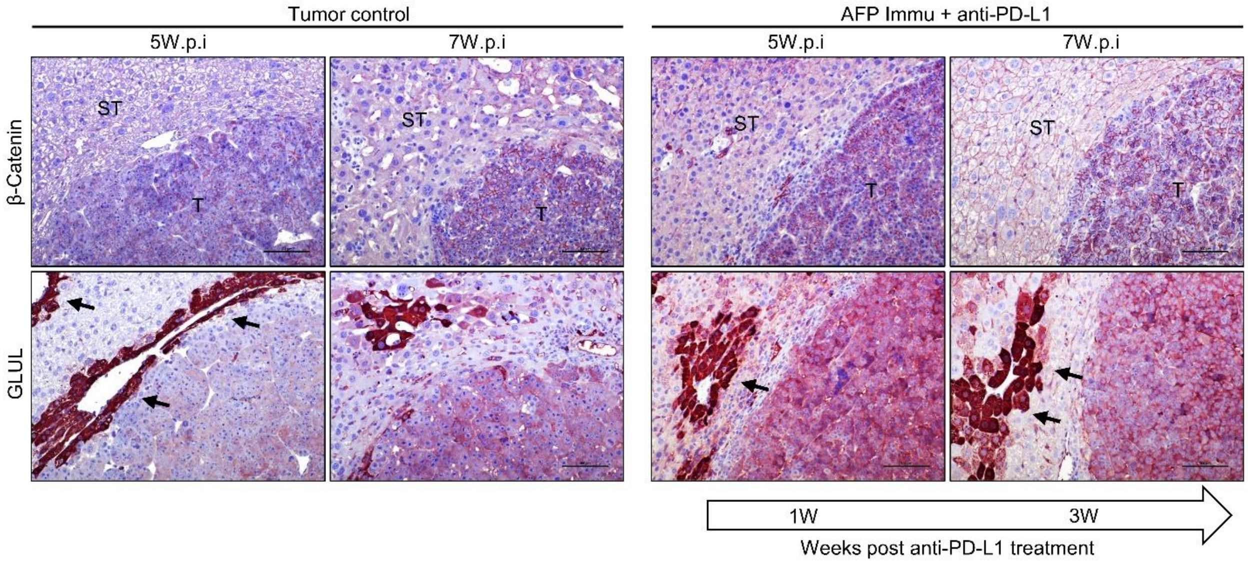

Supplementary Figure 28. Lack of β-Catenin pathway activation in remaining liver tumor lesions from c-MYC/Mcl1 mice subjected to AFP immunization and anti-PD-L1 administration. Immunohistochemistry showed the absence of nuclear accumulation of β-Catenin (a surrogate marker of its activation) in the tumor control lesions and those treated with AFP immunization and anti-PD-L1 combination therapy. In addition, levels of β-Catenin liver-specific target glutamine synthetase (GLUL) were equivalent in control and treated tumors, and significantly lower than those of hepatocytes surrounding the hepatic venules (indicated by arrows). Original magnification: 200X. Abbreviations: ST, surrounding tissue; T, tumor region; W.p.i, weeks post-injection.

Supplementary Table 1. Tumor nodules quantification of the Comb group

No.	Sex	Survival (Weeks)	Tumor nodules
1	F	7.71	0
2	M	8.14	12
3	F	10.57	2
4	M	11.00	8
5	M	12.14	2
6	F	14.29	1
7	M	15.57	1
8	M	25.00	0
9	F	25.00	0
10	F	25.00	0

Supplementary references

1. Poggio M, et al. Suppression of Exosomal PD-L1 Induces Systemic Anti-tumor Immunity and Memory. *Cell* 2019;177:414-27 e13.
2. Xu Z, et al. The mTORC2-Akt1 Cascade Is Crucial for c-Myc to Promote Hepatocarcinogenesis in Mice and Humans. *Hepatology* 2019;70:1600-13.
3. Cao Z, et al. MYC phosphorylation, activation, and tumorigenic potential in hepatocellular carcinoma are regulated by HMG-CoA reductase. *Cancer Res* 2011;71:2286-97.
4. Lu X, et al. YAP Accelerates Notch-Driven Cholangiocarcinogenesis via mTORC1 in Mice. *Am J Pathol* 2021;191:1651-67.
5. Wang H, et al. Distinct and Overlapping Roles of Hippo Effectors YAP and TAZ During Human and Mouse Hepatocarcinogenesis. *Cell Mol Gastroenterol Hepatol* 2021;11:1095-117.
6. Wu T, et al. clusterProfiler 4.0: A universal enrichment tool for interpreting omics data. *Innovation (N Y)* 2021;2:100141.
7. Ge SX, et al. iDEP: an integrated web application for differential expression and pathway analysis of RNA-Seq data. *BMC Bioinformatics* 2018;19:534.
8. Chen Z, et al. seq-ImmuCC: Cell-Centric View of Tissue Transcriptome Measuring Cellular Compositions of Immune Microenvironment From Mouse RNA-Seq Data. *Front Immunol* 2018;9:1286.
